# Colonic miRNA Expression/Secretion, Regulated by Intestinal Epithelial PepT1, Plays an Important Role in Cell-to-Cell Communication during Colitis

**DOI:** 10.1371/journal.pone.0087614

**Published:** 2014-02-19

**Authors:** Saravanan Ayyadurai, Moiz A. Charania, Bo Xiao, Emilie Viennois, Yuchen Zhang, Didier Merlin

**Affiliations:** 1 Department of Biology and Chemistry, Center for Diagnostics and Therapeutics, Georgia State University, Atlanta, Georgia, United States of America; 2 Veterans Affairs Medical Center, Decatur, Georgia, United States of America; Virginia Tech, United States of America

## Abstract

PepT1 is a member of the proton-oligopeptide cotransporter family SLC15, which mediates the transport of di/tripeptides from intestinal lumen into epithelial cells. MicroRNAs (miRNAs), a small noncoding RNAs (21–23 nucleotides), post-transcriptionally regulate gene expression by binding to the 3′-untranslated regions (UTRs) of their target mRNAs. Although the role of most miRNAs remains elusive, they have been implicated in vital cellular functions such as intestinal epithelial cells differentiation, proliferation, and apoptosis. In the present study, we investigated the effect of intestinal epithelial PepT1 expression on microRNA (miRNA) expression/secretion in the colons of control mice and in mice with experimentally induced colonic inflammation (colitis). The colonic miRNA expression was deregulated in both colitis and control mice but the deregulation of miRNA expression/secretion was specific to colonic tissue and did not affect other tissues such as spleen and liver. Intestinal epithelial PepT1-dependent deregulation of colonic miRNA expression not only affects epithelial cells but also other cell types, such as intestinal macrophages. Importantly, we found the miRNA 23b which was known to be involved in inflammatory bowel disease was secreted and transported between cells to impose a gene-silencing effect on recipient intestinal macrophages. Based on our data, we may conclude that the expression of a specific protein, PepT1, in the intestine affects local miRNA expression/secretion in the colon on a tissue specific manner and may play an important role during the induction and progression of colitis. Colonic miRNA expression/secretion, regulated by intestinal epithelial PepT1, could play a crucial role in cell-to-cell communication during colitis.

## Introduction

PepT1 is a di/tripeptide transporter [1]–[3] highly expressed in epithelial cells of the small intestine, and poorly expressed in the colon [4], [5]. However, colonic PepT1 expression is enhanced under conditions of chronic inflammation such as Inflammatory Bowel Disease (IBD) [4], [6], [7]. Recently, a polymorphism of the hPepT1 gene has been shown to be associated with IBD susceptibility, indicating a direct involvement of this transporter in IBD [8]. Also, it has been shown that PepT1 expressed in immune cells played an important role in promoting the immune response during experimentally induced colitis compared to the knock-out mice [9]. Recent study showed that IL-16 induces intestinal inflammation by up regulating PepT1 expression in puffer fish intestine and it subsequently transported the bacterial peptide fMLP which triggers the intestinal inflammation [7].

To specifically examine the role played by hPepT1 in intestinal epithelial cells (IECs) (including colonocytes) during intestinal inflammation, we generated a transgenic mouse model, in which hPepT1 was specifically expressed in IECs under the control of a villin promoter (villin-hPepT1 mice). Western blotting confirmed expression of hPepT1 in the colonocytes of transgenic mice (TG) but not those of wild-type (WT) mice [10]. The mice appeared healthy and exhibited normal body weight, breeding behavior, and general appearance. The morphology of the gastrointestinal tract as judged by histological examination appeared normal [10]. Immunohistochemical analysis verified hPepT1 membrane staining of colonocytes in villin-hPepT1 Tg mice, but not in WT mice [10]. In addition, apical membrane vesicles prepared from colonocytes of villin-hPepT1 mice, expressed significantly higher levels of functional hPepT1 compared to WT mice [10]. Together, the results clearly showed that villin-hPepT1 mice express hPepT1 in the colonic mucosa, which has also been observed in IBD patients [4], [6] and therefore represents an appropriate model in which the pathogenic role of hPepT1 in IBD may be explored.

To assess the effect of hPepT1 expression in colonocytes during inflammation, colitis was induced using dextran sulfate sodium (DSS) and TNBS [10]–[14]. Such treatment induced a more drastic weight loss in villin-hPepT1 mice with a higher clinical score compared to WT mice [10]. The severity of DSS-induced colitis in villin-hPepT1 mice was accompanied by a rise in inflammation, as noted by colonoscopy, with massive mucosal erythema and bleeding. DSS treatment increased the extent of histological damage in villin-hPepT1 mice as shown by almost complete crypt disruption and inflammatory infiltration throughout the mucosa and submucosa, consistent with the marked increase noted in colonic MPO activity compared to that of WT mice. Importantly, villin-hPepT1 animals also exhibited a marked increase in DSS-induced colonic levels of the pro-inflammatory cytokines such as IL-1β, IL-6, TNF-α and IFN-γ, compared to WT animals [10]. Our studies demonstrated that IEC-specific hPepT1 expression exacerbated the susceptibility of mice to DSS-induced colitis and reduced intestinal recovery and healing after induction of inflammation.

microRNA (miRNA), described in *Caenorhabditis elegans* in 1993 [15], [16] are 18 to 22 nucleotides in length [17] and negatively regulate target mRNAs by binding to their 3′- untranslated region (UTR) [18]–[20]. Each miRNA can target hundreds of mRNAs within a given cell type [21] and a single mRNA is the target of multiple miRNAs [22], [23]. Thus, miRNAs contribute to the regulation of over 30% of protein-coding genes [24]. MiRNAs have been shown to play important roles in various biological processes including immunity, cell proliferation and development [25]–[27] and intestinal epithelial cell differentiation [28], [29]. Many studies have examined global miRNA expression in health and disease conditions [17], [30]–[35]. Tissue specific miRNAs from mouse have already been investigated [36]. Recently, we have shown the tissue specific expression of CD98 on miRNA expression in the same tissue [37]. In the present study, we investigated the effect of intestinal epithelial PepT1 expression on miRNA expression in the colon of control mice and in mice experiencing inflammation.

## Materials and Methods

### Generation of hPepT1 transgenic mice

Homozygous villin-hPepT1 mice were previously generated [10] and FVB WT mice were used as controls. All animal procedures were approved by the Animal Care Committee of Emory University and Georgia State University and were conducted in accordance to the *Guide for the Care of Use of Laboratory Animals* from the US Public Health Service.

### Induction of colitis

Six week old villin-hPepT1 and FVB WT male mice were used for this study. Colitis was induced by the addition of 3% (w/v) dextran sodium sulfate (DSS; molecular weight 36,000–50,000 Da; MP Biomedicals, LLC, OH) to the drinking water [9]. Physical characteristics such as body weight and pro and anti-inflammatory cytokine profile were matched with previously reported [10]. The mice were humanly euthanized, and colon, spleen and liver tissue were processed further by extracting total RNA for microarray analysis and immunohistochemistry after 7 days of DSS treatment.

### RNA extraction and miRNA expression analysis by miRNA Microarray

**Figure 1:**
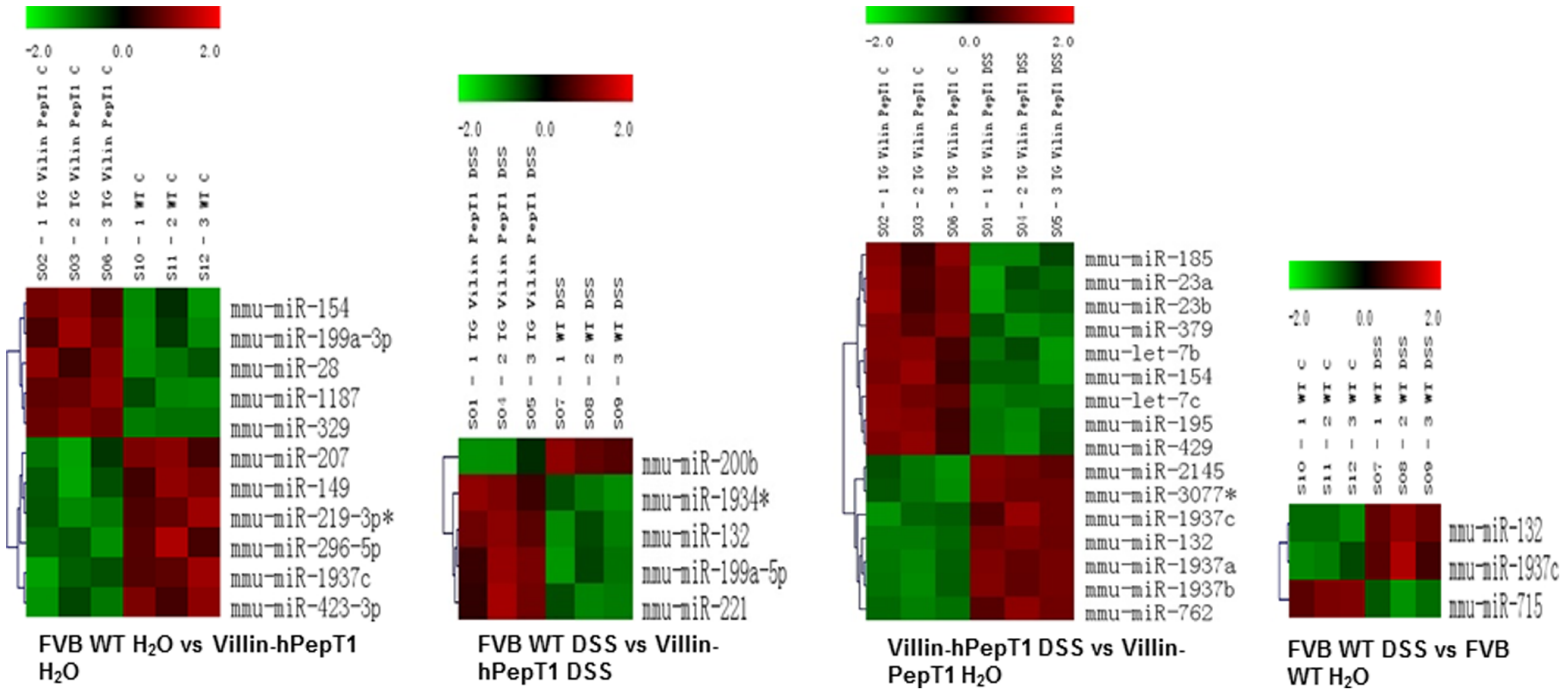
Clustering graph of miRNAs. microRNA microarray results demonstrated that villin-hPepT1 and FVB WT mice treated with and without DSS expressed different miRNA profiles. miRNAs expressed with a *p* value of <0.01 and signal >500.

Total RNA containing miRNA was extracted from the liver, spleen and colon of mice by RNeasy Plus Mini kit (Qiagen, Valencia, CA) according to the manufacturer's instruction given in Qiagen supplementary protocol for purification of miRNA from animal tissue. The yield and quality of RNA was verified. A size fractionation step with YM-100 Microcon filter was done which isolates nucleotides of ∼200 bp or less (LC Sciences, Houston, TX). MicroRNA microarrays were performed by using the μParaflo® microfluidic chip technology (LC Sciences). Probes for the arrays were developed using version 16 of the miRBase sequence database updated with 1145 miRNAs (http://www.lcsciences.com/applications/transcriptomics/mirna-profiling/mirna/). Data analysis for the arrays was performed using *t*-test, ANOVA and/or clustering analysis. We compared transcripts of FVB WT H_2_O vs villin-hPepT1 H_2_O, FVB WT H_2_O vs villin-hPepT1 treated with DSS, FVB WT H_2_O vs FVB WT DSS-treated and villin-hPepT1 H_2_O vs villin-hPepT1 DSS-treated (Fig. 1).

### Real-time RT-PCR analysis

cDNA was generated from the total RNAs isolated above using the NCode™ miRNA first-strand cDNA synthesis kit (Invitrogen, Carlsbad, CA) or Maxima first strand cDNA synthesis kit (Thermo Scientific, Glen-Burnie, MD) as previously described [38]. Levels of mature miRNAs expression were quantified by qRT-PCR using Maxima® SYBR Green/ROX qPCR Master Mix (Thermo Scientific, Pittsburgh, PA). The universal reverse primer provided in the NCode™ miRNA first-strand cDNA synthesis kit and the specific microRNA forward primers were used. Small RNA 234 or cel-mir-39 was used as housekeeping gene. Fold-induction was calculated using the Ct method as follows: ΔΔCt = (Ct_Target_−Ct_housekeeping_)_group 1_−(Ct_Target_−Ct_housekeeping_)_group 2_, and the final data were derived from 2^−ΔΔCT^. All primers used for qRT-PCR are described in Table S5.

### miRNA target prediction

To determine the potential target genes of detected miRNAs, three different miRNA target prediction algorithms were used: PicTar (http://pictar.mdc-berlin.de) [39], MicroCosm (http://www.ebi.ac.uk/enright-srv/microcosm/htdocs/targets/v5/) [40] and TargetScan (http://www.targetscan.org/) [41]. The Matchminer program (http://discover.nci.nih.gov/matchmi ner/index.jsp) [42] was then used to determine genes that were identified by at least two algorithms.

### DAVID gene functional annotation tool

DAVID (the Database for Annotation, Visualization and Integrated Discovery) is a bioinformatics resource developed by the Laboratory of Immunopathogenesis and Bioinformatics [43]. All tools in the DAVID Bioinformatics Resources aim to provide functional interpretation of large lists of genes derived from genomic studies, such as microarray and proteomics studies. DAVID can be found at http://david.niaid.nih.gov or http://david.abcc.ncifcrf.gov. The DAVID Functional Annotation Tool mainly provides typical batch annotation and gene-GO term enrichment analysis to highlight the most relevant GO terms associated with a given gene list. The *p*-values associated with each annotation term inside each cluster are used to rank their biological significance. Thus, the top ranked annotation groups most likely have consistently lower p-values for their annotation members.

The miRNAs predicted common protein targets from the Matchminer program were later analyzed. The functional annotation of each protein was analyzed through clusters formed by DAVID. Each cluster that scored an enrichment score of >1.0 were taken for analysis and the *p*-value of each annotation cluster verified.

### Western blot analysis

Approximately 1.0 cm piece of colon were homogenized in radioimmunoprecipitation assay buffer (RIPA) (150 mM NaCl, 0.5% sodium deoxycholate, 50 mM Tris-HCl, pH 8, 0.1% SDS, 0.1% Nonidet P-40) supplemented with protease inhibitors (Roche Diagnostics, Indianapolis, IN) on ice. The homogenates were centrifuged at 12,000 rpm for 10 min at 4°C. Total cell lysates were resolved on 10% polyacrylamide gels (Bio-Rad, Hercules, CA) and transferred to nitrocellulose membranes (Bio-Rad). Membranes were then probed with Marcksl-1 (Proteintech, 10002-2-AP, Chicago, IL) and beta-tubulin (Sigma- Aldrich, T8328, St Louis, MO). After washes, membranes were incubated with appropriate horseradish peroxidase-conjugated secondary antibody (GE Healthcare Biosciences, Pittsburgh, PA), and blots were detected using the Enhanced Chemiluminescence Detection kit (GE Healthcare Biosciences).

### Immunohistochemistry

Paraffin-embedded tissue sections (5-µm) were deparaffinized in xylene and rehydrated in an ethanol gradient. Epitope retrieval was done by treating with 10 mM sodium citrate buffer (pH 6.0) and 10 mM citric acid (4∶1 ratio) at 120°C for 10 min in a pressure cooker. Sections were blocked with 5% goat serum and then incubated with Marcksl-1 (1∶100 dilution) overnight at 4°C. After washing with phosphate buffered saline (PBS), sections were incubated with Alexa-Fluor 568 phalloidin (Invitrogen, 1∶5000) and horseradish peroxidase-conjugated secondary antibody for 45 minutes at room temperature in the dark. Sections were mounted in mounting medium with 4′, 6-diamidino-2-phenylindole (DAPI) (Vector Laboratories, Burlingame, CA) and cover slipped. Images were acquired using an Olympus microscope equipped with an Hamamatsu Digital Camera ORCA-03G.

### Secreted miRNA in colon culture

DSS treated villin-hpepT1 and FVB WT along with H_2_O control mouse colons were cut and washed three times in sterile PBS and cultured in RPMI media without serum at 37°C for 24 hrs. The supernatant was collected and centrifuged to sediment the tissue particles. Cel-miR-39 was used as an invariant control for the conditioned medium RNA extraction and qRT-PCR analysis as described earlier [44], [45]. 10 µL of 0.1 nM cel-miR-39 was added to each 1 mL aliquot of supernatant and vortexed for 30s. miRNAs extraction was performed using the Norgen Biotek Exosome RNA extraction kit (Norgen Biotek, Ontario, Canada) according to the manufacturer's instructions and the RNA yield was verified. miRNA profiles from the conditioned medium or blank medium were assessed by qRT-PCR and further confirmed by Signosis miRNA expression plate assay according to the manufacturer's protocol (Signosis Inc, Sunnyvale, CA) and chemiluminescence was measured in a Biotek synergy 2.0 microplate reader (Biotek, Winooski, VT).

### miRNA uptake experiment in macrophages

Although many miRNA's up or down regulated in our study, it has been reported that miRNA 23a and miRNA 23b have a potential roles in ulcerative colitis and Crohn's disease in patients [46]. Therefore, we chose miRNA 23b for further study. MiRNA 23b uptake by macrophages was assessed using Cy3 labeled dsRNA 23b (5′-Cy3 labeled antisense strand) (Integrated DNA Technologies, Coralville, IA). 3×10^4^ macrophage (RAW-264.7) cells were grown in 6 chamber BD falcon culture slides (BD Bioscience, Bedford, MA) at 37°C with 5% CO_2_ for 24 h_._ The Cy3 labeled miRNA 23b at 50 nM concentration was added into the culture medium and incubated at 37°C with 5% CO_2_ for 24 h. Cells were washed with PBS and stained with Alexa Fluor 488 phalloidin (Invitrogen) and vectashield mounting medium with DAPI (Vector Laboratories, Burlingame, CA) and the slides were examined under microscope for miRNA 23b uptake by macrophages.

### Targeting Marcksl-1 3′UTR by miRNA 23b in macrophages

To examine whether miRNA-23b directly targets the 3′UTR of Marcksl-1 mRNA, Marcksl-1 3′UTR was cloned downstream in pEZX vector with neomycin/kanamycin selection with a luciferase reporter gene (Luc-Marcksl-1 3′UTR) (Genecopoeia, Rockville, MD). Precursor miRNA-23b cloned in CMV promoter with ampicillin selection (Genecopoeia, Rockville, MD). The constructs were transfected in XL gold ultracompetent cells (Agilent Technologies, Santa Clara, CA) and selected with appropriate antibiotic selection media. The plasmid extraction was done using Qiagen Medi-plasmid extraction kit (Qiagen, Valencia, CA). The plasmid concentration and the constructs position were verified using restriction enzymes. RAW-264.7 cells were grown in 6 well plate (3.3×10^5^) and Luc-Marcksl-1 3′UTR construct was co-transfected with miRNA-23b precursor. As controls, Luc-Marcksl-1 3′UTR and miRNA-23b precursor constructs alone transfected in RAW 264.7 cells using Lipofectamine 2000 (Life Technologies, Grand Island, NY) in Opti-MEM reduced serum medium (GIBCO, Life Technologies, Grand Island, NY). Only RAW 264.7 cells used as negative control. After 4 h of transfection, serum-free DMEM medium was added (cellgro, Manassas, VA). Transfected cells were incubated at 37°C for 24 h with 5% CO_2_. After incubation, the cells were washed with sterile PBS and luciferase assay was carried out according to the manufactures protocol (Genecopoeia, Rockville, MD). The targeting of miRNAs Marcksl-1 3′UTR was then assessed by measuring luciferase activity in these cells.

### Statistical analysis

Values were expressed as means ± standard error of mean (SEM). Statistical analysis was performed using unpaired two-tailed *t*-test by GraphPad Prism 5 software. *p*<0.05 were considered statistically significant.

## Results

### Differential effects of colonic PepT1 expression on colonic miRNA synthesis

**Figure 2:**
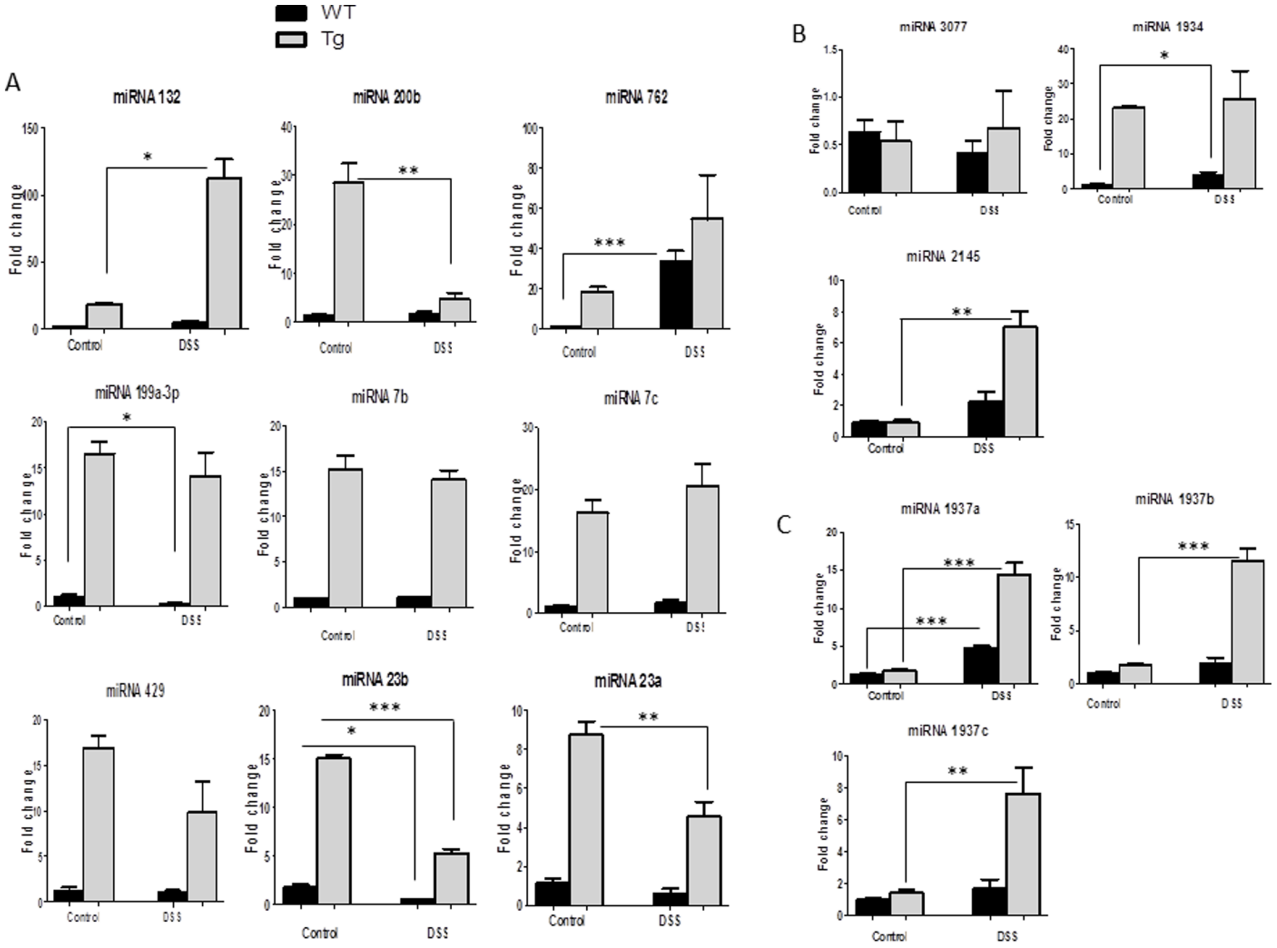
Colonic miRNA expression levels are modulated in villin-hPepT1 and FVB WT mice with and without DSS. miRNAs expressed with the a *p*-value of <0.01 and signal >500 in microRNA microarray were analyzed by qRT-PCR (Fig. 2A). miRNA 3077, miRNA 1934, miRNA 2145 were not associated with any target mRNAs so these three miRNAs were excluded from further analysis (Fig. 2B) and the mature mmu-miR-1937a, mmu-miR-1937b, and mmu-miR-1937c are fragments of tRNA (www.mirbase.org) so we eliminated these from further analyses (Fig. 2C). Difference in miRNA expression noted as * *p*<0.05, ** *p*<0.001 and *** *p*<0.0001. Values represent means ± SEM of n = 6/group.

The expression of colonic miRNA transcripts was compared between WT and villin-hPepT1 mice either treated with DSS, or not, at the significance levels of *p*<0.01, *p*<0.05, and *p*<0.1 (Table S1). Although large numbers of miRNAs were differentially expressed, many yielded low signal strengths (<500). These miRNAs and those for which the differential *p*-values greater than 0.01, were excluded from analysis (Fig. 1). Of the 15 miRNAs that met our stringent criteria, 3 were discarded: the mature miRNAs, mmu-miR-1937a, mmu-miR-1937b, and mmu-miR-1937c are in fact fragments of tRNA (www.mirbase.org) (Fig. 2C). The remaining 12 miRNAs with differential *p-*values<0.01 and of signal strengths >500 were further analyzed by qRT-PCR to determine fold changes (Fig. 2A and Fig. 2B). The data were compared with the differential expression levels detected upon microarray analysis (Table S1). Out of 12 microRNAs, 10 were differentially expressed, with statistical significance, when non-treated WT and villin-hPepT1 mice were compared (the Student's *t*-test values were 0.001–0.04 with *p*-values<0.01; the fold changes yielded upon qRT-PCR analysis were comparable).

### Intestinal epithelial PepT1 expression upregulates basal colonic miRNA expression

**Figure 3:**
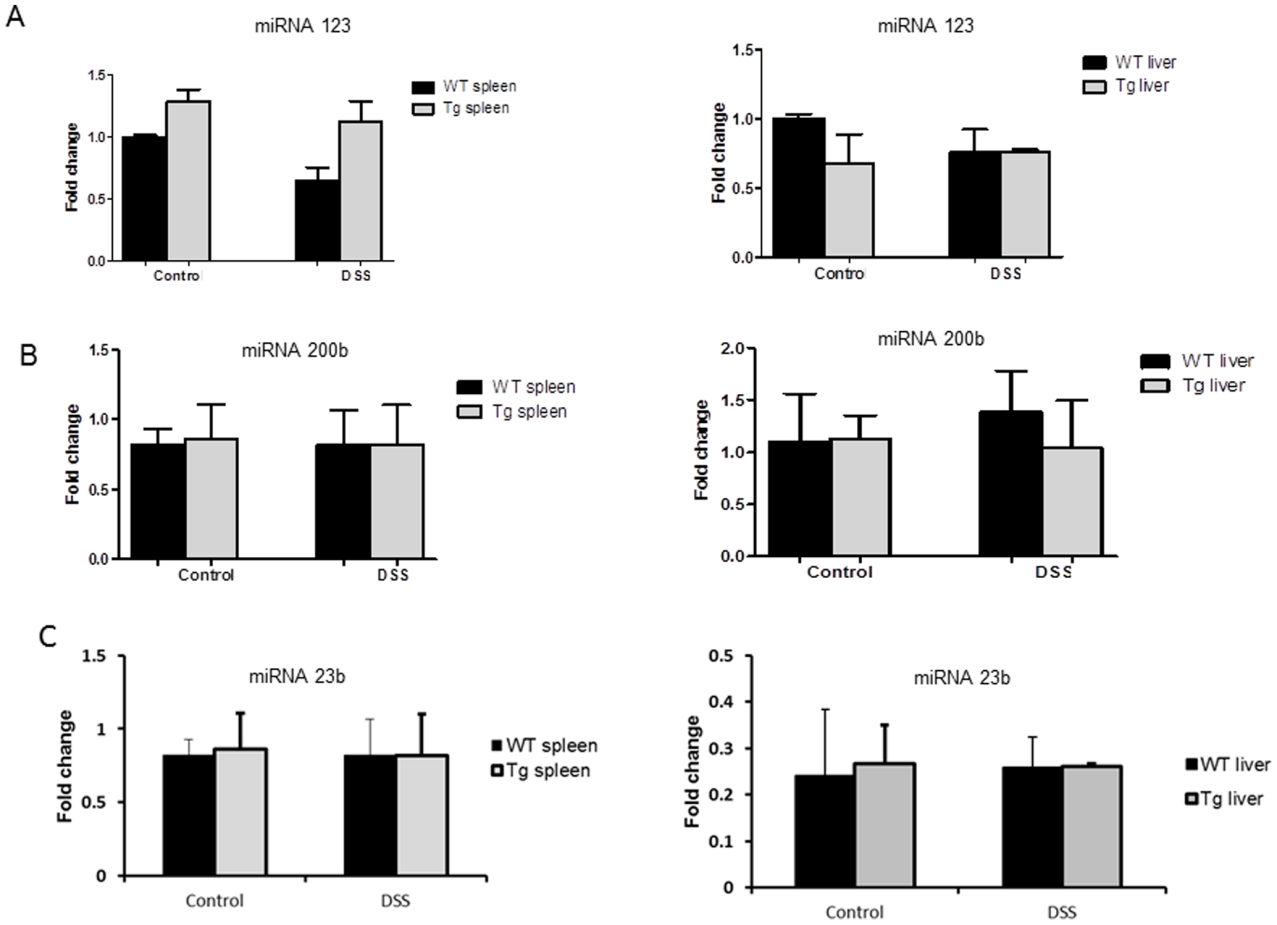
hPepT1 regulates miRNA 132, miRNA 200b and miRNA 23b expression in colon- specific manner. Upon exposure to DSS or normal drinking water, intestinal epithelial PepT1 overexpression did not affect the expression level of miRNA 132 (Fig. 3A), miRNA 200b (Fig. 3B) and miRNA 23b (Fig. 3C) in liver or spleen. Values represent means ± SEM of n = 6/group.

First, we investigated the effect of intestinal epithelial PepT1 expression on colonic (epithelial and non-epithelial cells) miRNA expression in untreated mice. The expression levels of the 12 relevant miRNAs, as calculated by qRT-PCR, were in agreement with the miRNA microarray data (Fig. 2A and 2B). A total of 10 colonic miRNAs (miRNA 132, 200b, 762, 199a-3p, 7b, 7c, 429, 23b, 23a, and 1934) were upregulated in villin-hPepT1 mice compared to WT animals. In contrast, miRNAs 3077 and 2145 were expressed at similar levels in both mouse strains (Fig. 2B). Importantly, we found that intestinal epithelial PepT1 overexpression did not affect the expression level of any of these 12 miRNAs in other tissues, including the liver and spleen. We herein represented miRNA 132 (Fig. 3A), miRNA 200b (Fig. 3B) and miRNA 23b (Fig. 3C) in liver and spleen respectively. Thus, overexpression of intestinal epithelial PepT1 specifically upregulated 10 colonic miRNAs. Next, we identified the mRNAs targeted by these miRNAs using Pictar, MicroCosm and Targetscan algorithms. Matchminer program was then employed to identify target genes detected by at least two of the former algorithms. miRNA 3077, miRNA 1934 and miRNA 2145 were not found to target any mRNA and these three miRNAs were excluded from further analysis (Fig. 2B). The remaining nine miRNAs potentially regulated the expression of 704 genes (Table S2).

DAVID functional annotation clustering of villin-hPepT1 and WT at the basal level identified a total of 30 clusters of 453 genes; 161 genes were not part of any of these clusters. The most enriched functional groups (score >1.0) included eleven clusters of genes that were potentially downregulated. These genes are involved in transcription regulation, protein biosynthesis, cell division, endocytosis and calcium binding domains (Table 1) and (Table S3).

**Table 1:** DAVID functional annotation clustering of villin-hPepT1 and FVB WT at the basal level.

Annotation cluster	Enrichment score	Enriched functional group gene annotation/function	Total gene count
1	4.77	Transcription and Transcription regulation	144
		Nucleus	128
		DNA – binding	54
2	3.31	Isopeptide bond	18
		Ubl conjugation	27
		Cross-links: glycyl lysine isopeptide (Lys-Gly) (interchain with G-Cter in SUMO)	10
3	2.17	Nucleotide-binding	64
		atp-binding	51
		Transferase	53
		Kinase	28
		Domain: protein kinase	22
		Binding site: ATP	24
		Nucleotide phosphate-binding region: ATP	35
		Serine/threonine-protein kinase	17
		Active site: protein acceptor	24
4	2.05	Golgi apparatus	28
		Topological domain: Lumenal	20
		glycosyltransferase	11
		Signal-anchor	17
5	2	Domain:W2	5
		Initiation factor	4
		Protein biosynthesis	5
6	1.83	Repeat: LDL-receptor class B1–20	71
		Repeat: LDL-receptor class A1–8	27
		Domain: EGF-like1–8	45
		Domain: EGF-like 2;calcium binding	3
		Endocytosis	6
		Short sequence modif: Endocytosis signal	3
7	1.56	Nucleotide binding	6
		P-Loop	6
		GTP binding	4
8	1.37	Calcium and calcium binding region 1–2	41
		Domain: EF-hand 1–3	24
9	1.35	Cell division	13
		Cell cycle	19
		Mitosis	8
10	1.12	Short sequence motif: DEAD box	4
		Short sequence motif: Q box	4
		Domain: Helicase C-terminal	6
		Domain: Helicase ATP binding	6
		Helicase	6
11	1.1	Domain: WW 1–3	9

The common proteins were clustered into 30 groups, but based on enrichment score >1.0. 11 groups were considered to be the potential targets with a total of 1100 genes.

### Intestinal epithelial PepT1 expression deregulates colonic miRNA expression under induced colitis

To investigate the effect of intestinal epithelial PepT1 expression on colonic miRNA expression after induction of colitis, villin-hPepT1 and WT mice were treated with DSS (given in drinking water) for 7 days and the levels of the nine colonic miRNAs described above were measured. As shown in Fig. 2A, DSS did not significantly affect the expression levels of colonic miRNAs 762,199a-3p, 429, 7b, or 7c in Tg mice, nor those of miRNAs 132, 200b, 7b, 7c, 429, or 23a in WT mice, compared to the levels seen in untreated WT or Tg mice. In contrast, colonic miRNA 762 was up-regulated and colonic miRNA 199a-3p and miRNA 23b significantly down-regulated after DSS treatment in WT mice (Fig. 2A). Interestingly, colonic miRNAs 200b, 23b, and 23a were down-regulated, and 132 up-regulated in villin-hPepT1 mice after DSS-treatment (Fig. 2A). However, the expression levels of all nine miRNAs were significantly higher in DSS-treated villin-hPepT1 compared to DSS treated WT mice (Fig. 2A). The miRNA fold changes observed in microarray and verified by qRT-PCR are listed in Table 2.

**Table 2:** miRNAs differentially expressed after DSS treatment in colon.

Upregulated miRNAs		Downregulated miRNAs	
Name	Fold change	Name	Fold change
mmu-miR-132	7.81	mmu-miR-200b	−6.05
mmu let-7c	1.27	mmu-miR-429	−1.71
		mmu-miR-23b	−2.85
		mmu-miR-23a	−1.92

miRNA microarray with the fold change (up-regulated and/or down-regulated) listed in the table.

Upon DSS exposure, intestinal epithelial PepT1 overexpression did not affect the level of miRNA expression in other tissues such as liver or spleen (Fig. 3A, 3B and 3C). These results suggest that the mRNAs targeted by the nine colonic miRNAs could be differentially expressed in mice over expressing intestinal epithelial PepT1 compared to animals in which intestinal epithelial PepT1 is not expressed or expressed at low basal levels.

DAVID functional annotation clustering of villin-hPepT1 and FVB WT upon DSS treatment identified a total of 15 clusters of 196 genes; 83 genes were not part of any of these clusters (Table S4). The most enriched functional groups identified 4 clusters of genes involved in transcription, ion channel and transport, transcription, calcium binding and DNA binding (Table 3).

**Table 3:** DAVID functional annotation clustering of villin-hPepT1 and FVB WT treated with DSS.

Annotation cluster	Enrichment score	Enriched functional group gene annotation/function	Total gene count
1	2.96	Transcription and Transcription regulation	68
		Nucleus	59
		Activator	14
		DNA – binding	26
2	1.92	Isopeptide bond	9
		Ubl conjugation	13
		Cross-links: glycyl lysine isopeptide (Lys-Gly) (interchain with G-Cter in SUMO)	5
3	1.79	Calcium and calcium-binding region 1 and 2	26
		Domin:EF-hand 1, 2 and 3	17
4	1.12	Potassium and Potassium transport	10
		Potassium channel	4
		Voltage-gated channel	5
		Ion transport	9
		Ionic channel	5

The common proteins obtained from the matchminer software were clustered in to 15 groups but based on enrichment score >1.0, a total of 4 groups were considered to be the potential targets with a total of 270 genes.

### Deregulation of colonic miRNAs occurs not only in epithelial cells but also in immune cells such as macrophages

**Figure 4:**
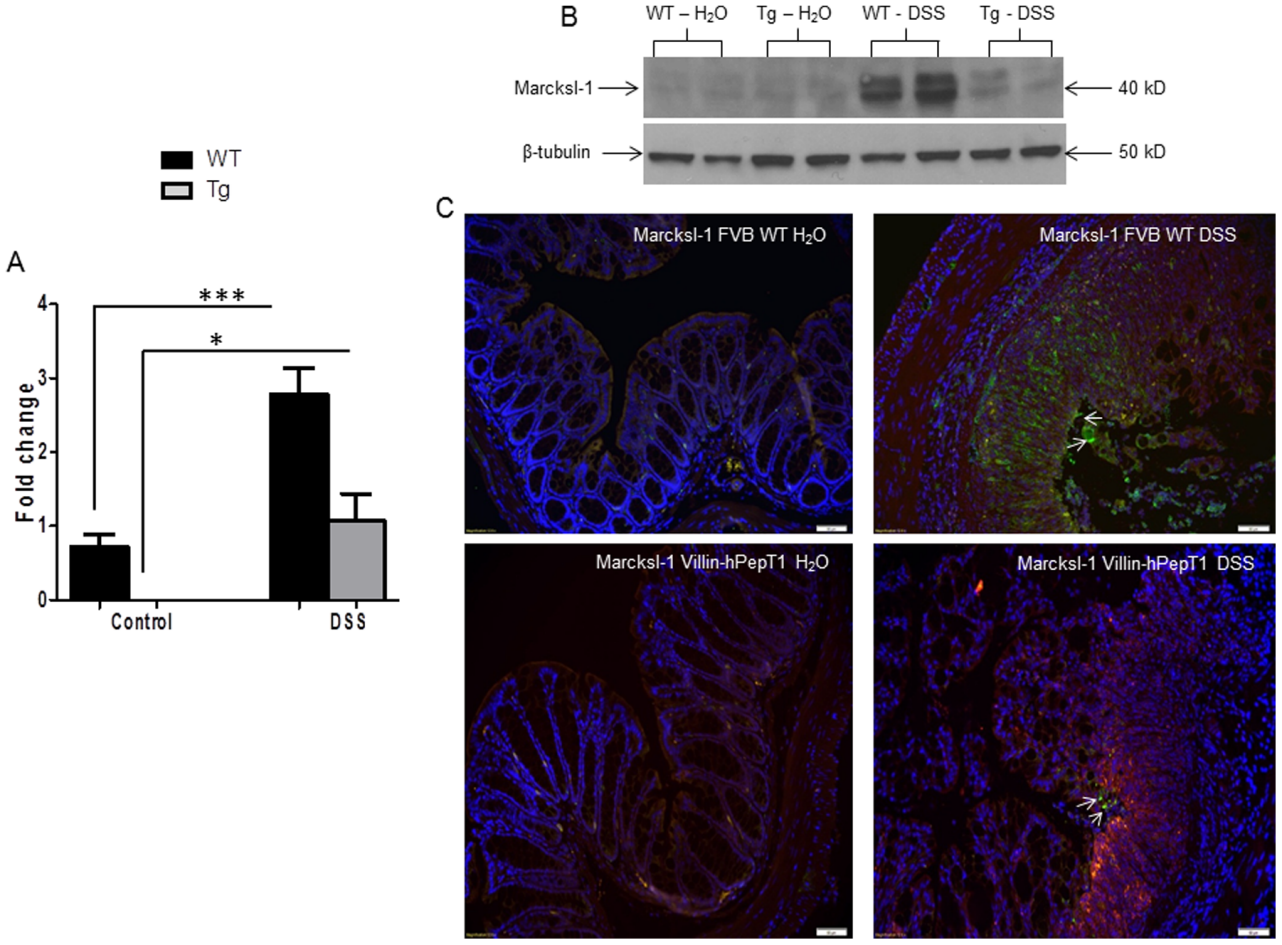
Marcksl-1 is regulated by colonic miRNA 23b in DSS-treated FVB WT mice. Marcksl-1 mRNA fold level was quantified by qRT-PCR (Fig. 4A). Western blot demonstrated protein expression was observed at high level in WT DSS-treated mice and at low level Marcksl-1 and/or not at all in villin-hPepT1 DSS-treated mice compared to their respective control groups. Beta tubulin, 50 kDa was used as a loading control (Fig. 4B). Immunofluorescence staining of Marcksl-1 protein expression (green) in FVB WT DSS mice compared to villin-hPepT1 DSS treated mice (Fig. 4C). Marcksl-1 (FITC - green), nuclear staining (DAPI - blue) and F-actin staining (TRITC - red) and separate pictures were taken at 40× for each filter and merged. Values represent means ± SEM of n = 6/group. *p<0.05 and ***p<0.0001. Scale bar = 50 µm.

The miRNA expression analysis was performed with the whole colon RNA, therefore the observed differences in colonic miRNA expression likely reflect changes in both epithelial and non-epithelial (*i.e*., immune) cells. For this purpose, we looked at the mRNA and protein expression of a non-epithelial protein macrophage myristoylated alanine-rich C kinase substrate (Marcksl-1), a potential target gene of miRNA 23a and 23b (Table S2). We chose miRNA 23b for our study as it was previously shown to be involved in chronically active Crohn's disease [47], and active ulcerative colitis [46]. In addition, Marcksl-1 is specifically expressed in macrophages [48] and has been associated with gastrointestinal and inflammatory disease [49], apoptosis and immune response [50], [51]. As shown in Fig. 4A and 4B, qRT-PCR and western blot analysis of Marcksl-1 mRNA and protein levels upon DSS treatment were up-regulated in WT mice compared to villin-hPepT1 and were in agreement with colonic miRNA 23b expression levels after DSS treatment (Fig. 2A). The anti-Marcksl-1 immunofluorescence from the colon tissue of WT and villin-hPepT1 mice with and without DSS treatment also showed the WT DSS-treated mice had higher protein accumulation compared to DSS-treated villin-hPepT1 mice (Fig. 4C).

### Intestinal epithelial PepT1 expression up-regulates the secretion of colonic miRNA 23b

**Figure 5:**
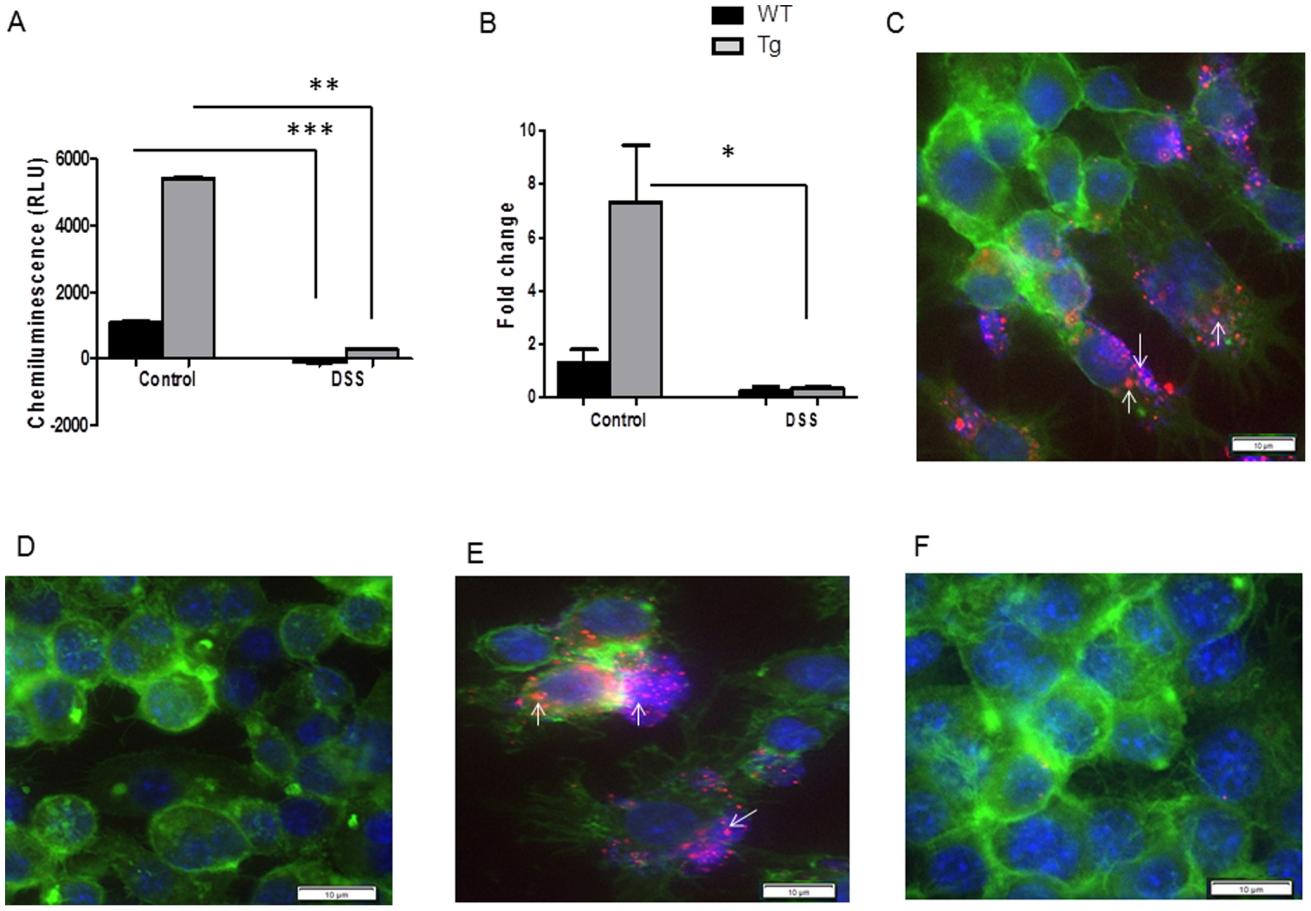
Secretory miRNA in colon culture medium. miRNA 23b in conditioned medium was measured by miRNA plate assay chemiluminescence method (Fig. 5A) and qRT-PCR (Fig. 5B). Cy3 labelled miRNA 23b uptake by macrophage (Fig. 5C). miRNA 23b uptake at 4°C (Fig. 5D), miRNA 23b uptake at pH 7.0 (Fig. 5E) and pH 6.5 (Fig. 5F). F-actin (FITC - green), nuclear staining (DAPI - blue) and Cy3 labelled miRNA 23b (TRITC -filter setting used) are stained and separate pictures were taken at 60× for each filter and merged respectively. Values represent means ± SEM of n = 6/group. *p<0.05, **p<0.001 and ***p<0.0001. Scale bar = 10 µm.

An analysis of miRNA 23b secretion was performed in colon cultures from villin-hPepT1 and FVB WT mice with or without DSS treatment. Secreted miRNA 23b in the colon culture medium was assessed using a miRNA plate assay method and qRT-PCR. The levels of secreted miRNA 23b were higher in colonic tissues from villin-hPepT1 control mice compared to colonic tissues from FVB WT mice (Fig. 5A and 5B). Notably, DSS treatment significantly reduced the levels of secreted miRNA 23b in the colon culture medium coming from both WT and villin-hPepT1 control mice (Fig. 5A and 5B). These results showed that an increase in intestinal epithelial PepT1 expression enhanced the secretion of miRNA 23b from colonic tissue. Interestingly, up-regulated levels of colonic miRNA 23b secretion induced by intestinal epithelial hPepT1 expression remained higher in colonic tissue expressing hPepT1 compared with colonic tissue with no or low PepT1 expression (Fig. 5A and 5B).

### Colonic miRNA 23b is actively up-taken by colonic macrophages

Next, we investigated the possible uptake of secreted colonic miRNA 23b by colonic macrophages. To examine this possibility, we labeled the 5′-end of dsmiRNA 23b with Cy3 and assessed its uptake behavior into macrophages. As shown in Figure 5C, Cy3-labeled miRNA 23b was effectively taken up by macrophages incubated in the presence of 50 nM Cy3-labeled miRNA 23b for 24 hours at 37°C and pH 7.2. In addition, miRNA 23b uptake by macrophages was inhibited at 4°C (Fig. 5D), indicating that it is an active energy-dependent process. We also observed that miRNA 23b uptake by macrophages occurs at pH 7.0, 7.2 or 7.8 (Fig. 5E), but not at pH 6.0 or 6.5 (Fig. 5F). This latter observation is consistent with an endocytosis-mediated uptake mechanism. Taken together, our results demonstrated that secreted colonic miRNA 23b may be actively taken up by macrophages via an endocytosis/phagocytosis-mediated mechanism.

### MiRNA 23b directly targets the Marcksl-1 3′UTR in macrophages

**Figure 6:**
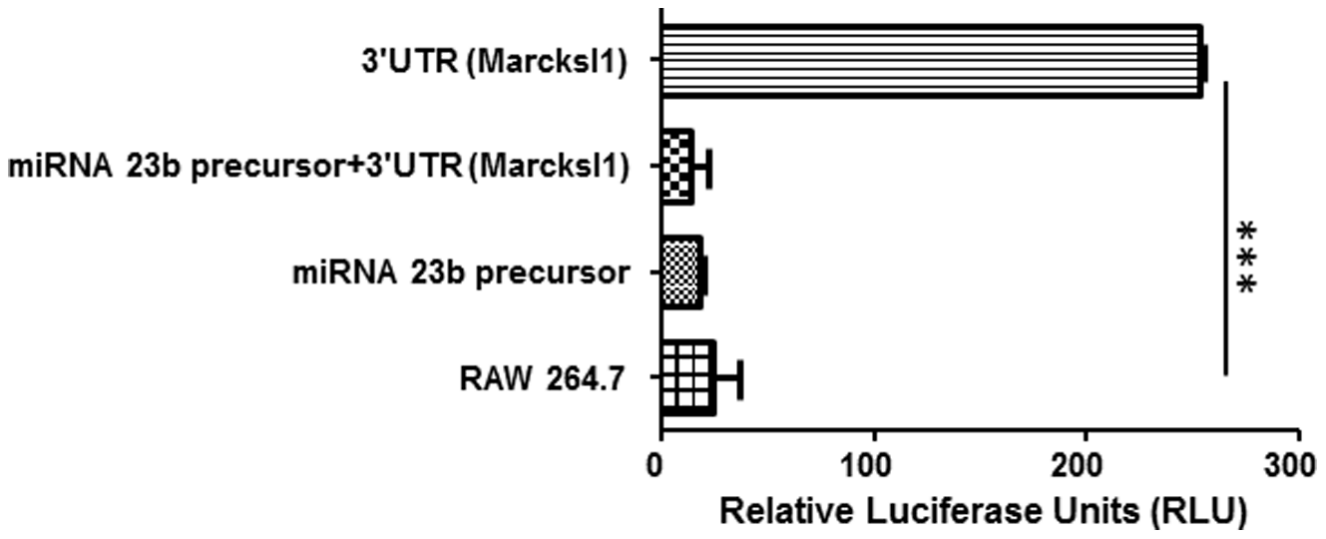
MiR-23b directly targets the Marcksl-1 3′UTR in macrophages. Marcksl-1 3′UTR was cloned into a luciferase reporter gene (Luc-Marcksl-1 3′UTR), and transfected into RAW 264.7 cells in the presence or absence of miRNA 23b precursor. The targeting of miRNAs to Marcksl-1 3′UTR was then assessed by measuring luciferase activity in these cells. The miRNA 23b co-transfected with Luc-Marcksl-1 3′UTR significantly reduced luciferase activity in macrophage cells after 24 h of transfection. In contrast, Luc-Marcksl-1 3′UTR had an increase of luciferase activity. Values represent means ± SEM of n = 3 and ***p<0.0001.

To examine whether miRNA 23b directly targets the 3′UTR of Marcksl-1 mRNA, Marcksl-1 3′UTR was cloned into a luciferase reporter gene (Luc-Marcksl-1 3′UTR), and this construct was transfected into RAW 264.7 cells in the presence of miRNA 23b precursor or alone. The targeting of miRNAs to Marcksl-1 3′UTR was then assessed by measuring luciferase activity in these cells. As shown in Fig. 6, miRNA 23b co-transfected with Luc-Marcksl-1 3′UTR significantly reduced luciferase activity in macrophage cells after 24 h of transfection. In contrast, Luc-Marcksl-1 3′UTR luciferase activity remained unchanged. The reduction of luciferase activity indicated that miRNA 23b targeted Marcksl-1 in its 3′UTR.

## Discussion

It has been recently reported that specific human colonic miRNAs are differentially expressed in IBD [30]–[32]. However, the mechanism underlying this deregulation has not been studied. To examine the effects of colonic PepT1 expression on colonic miRNA expression in untreated mice and in mice with DSS-induced colitis, we used a Tg mouse strain specifically overexpressing hPepT1, (villin-hPepT1) in colonic epithelial cells (IECs) driven by the villin promoter [10]. Our results show that overexpression of PepT1 in IECs alters the profile of miRNA expression in colonic cells, including both epithelial and non-epithelial cells such as intestinal macrophages. In contrast, the expression of miRNAs in non-colonic tissues was not affected in villin-hPepT1 mice. This latter observation demonstrates that the expression of intestinal epithelial PepT1 specifically deregulates the expression of colonic miRNAs. Our results demonstrate that expression of a specific protein involved in IBD (PepT1) may deregulate colonic miRNAs under conditions of both active (DSS) and non-active (without DSS) inflammation. Interestingly, these changes were initiated by intestinal epithelial PepT1 expression. These data predict that in a disease state such as intestinal inflammation, IECs over-expressing PepT1 can communicate directly with IECs and/or indirectly through cell/cell interactions; hence, epithelial PepT1 affects miRNA expression in other cells that are in contact with IECs. For example, recent studies have shown that extracellular vesicles contain functional mRNA, miRNA, and DNA molecules that can be taken up by target (acceptor) cells, indicating that these vesicles are a main mechanism for cell-to-cell communication [52]–[56]. It has recently been demonstrated by Kosaka *et al*. and other groups, that a tumor-suppressive miRNA is secreted and transported between cells to impose a gene-silencing effect on recipient cells [44], [45], [57]. miRNAs have been found in extracellular space such as plasma, urine, saliva and constitute a form of cell-cell communication [58]–[61]. Although we do not understand the cellular machinery responsible for the secretion of miRNAs, it is known that they are packaged into exosomes, microvesicles and apoptotic bodies by a broad range of cell types [62]–[64]. Intriguingly, most extracellular miRNAs are found outside of any lipid-containing vesicle. They are rather associated with RNA-binding proteins (*e.g*., Argonaute 1 and 2), which may help protect them from the abundant nucleases found in the extracellular space [65]. It has been previously shown by others that under laboratory conditions colon tissues can be cultured and shown to maintain viability [66]–[68]. Here, we demonstrated that colonic tissue secretes miRNA 23b and this secretion is regulated by the expression of intestinal epithelial hPepT1. Interestingly, we have demonstrated that miRNA 23b is actively taken up by macrophages, presumably through endocytosis. In addition, we have demonstrated that miRNA 23b directly targets the Marcksl-1 3′UTR in macrophages. Our results suggest that IECs may secrete miRNAs that are taken up by other cell types such as macrophages, resulting in regulation of recipient cell target genes such as Marcksl-1.

It has been previously shown that miRNA 23a and miRNA 23b are involved in active ulcerative colitis [46] and chronically active Crohn's disease [32]. These results are consistent with the observed colonic expression of miRNA 23a and 23b in WT and villin-hPepT1 mice with or without DSS treatment (Fig. 2A). Macrophage expression of Marcksl-1, one of the predicted target genes of miRNA 23a and 23b (Table S2), has been suggested to protect the intestine against inflammation [49]. Interestingly, we have observed that, although Marcksl-1 is specifically expressed in macrophages [48], its mRNA and protein expression profile are altered during disease conditions in hPepT1 Tg mouse model. We have indeed demonstrated that Marcksl-1 expression is increased to a lesser extent in intestinal macrophages during DSS-induced colitis in villin-hPepT1 mice than in WT mice. This observation may suggest that Marcksl-1 expression in intestinal macrophages is repressed by the more prominent elevation of miRNA 23b in inflamed villin-hPepT1 mice compared with inflamed WT mice. In addition, the low expression of Marcksl-1 in intestinal macrophages in villin-hPepT1 mice under colitis conditions may explain the greater degree of intestinal inflammation in these mice that over-express PepT1 in IECs.

Herein, we have shown that miRNA 23b expression levels were higher in the colonic tissues (but not other tissues) of villin-hPepT1 mice compared to WT mice, regardless of DSS treatment. Thus, overexpression of intestinal epithelial PepT1 specifically up-regulated colonic miRNA 23b in villin-hPepT1 mice. MiRNA-23b expression was lower in DSS-treated villin-hPepT1 mice compared to untreated villin-hPepT1 mice. However, miRNA 23b expression was higher in DSS-treated villin-hPepT1 mice compared to DSS-treated WT mice. The latter observation suggests that the level of miRNA 23b expression in DSS-treated villin-hPepT1 mice (but not that in DSS-treated WT mice) could be sufficient to target and repress Marcksl-1 expression, which is lower in DSS-treated villin-hPepT1 mice than in DSS-treated WT mice (Figure 4B and 4C). However, miRNA-23b over-expression in untreated villin-hPepT1 mice does not appear to affect Marcksl-1 expression, which is extremely low in non-inflamed intestine (Figure 4C). Collectively, these results show that local expression of a specific colonic protein may affect colonic miRNA expression/secretion. In addition, colonic miRNA expression/secretion, regulated by intestinal epithelial PepT1, may play an important role in cell-to-cell communication during colitis. Together, these results suggest that miRNA-23b expression may play a role in regulating Marcksl-1 during intestinal inflammation.

## Supporting Information

Table S1 **List of miRNAs differentially expressed in microRNA microarray.** The transcripts listed in the table having a statistically significant *p*-value of *p*<0.1 and having a signal of >500.
(PDF)

Table S2 **The potential mRNA targets for the nine selected miRNA were obtained from 3 different software Pictar, MicroCosm and Targetscan.** The common protein list was obtained from matchminer program by determining the genes that were identified by at least two algorithms. A total of 704 genes were potentially regulated by the 9 miRNA revealed from this software. These miRNA were sub-divided into two groups based on their expression levels in either FVB WT or villin-hPepT1 mice upon DSS treatment.
(XLS)

Table S3 **DAVID functional annotation clustering of villin-hPepT1 and FVB WT at basal level.** The common obtained from the matchminer software proteins were clustered into 30 groups.
(PDF)

Table S4 **DAVID functional annotation clustering of villin-hPepT1 and FVB WT mice treated with DSS.** The common proteins obtained from the matchminer software were clustered into 15 groups.
(PDF)

Table S5 **List of primers used in this study.**
(PDF)

## References

[1] Dalmasso G, Nguyen HT, Charrier-Hisamuddin L, Yan Y, Laroui H, et al (2010) PepT1 mediates transport of the proinflammatory bacterial tripeptide L-Ala-{gamma}-D-Glu-meso-DAP in intestinal epithelial cells. Am J Physiol Gastrointest Liver Physiol 299: G687–696. 20558765 10.1152/ajpgi.00527.2009 PMC2950691

[2] Buyse M, Tsocas A, Walker F, Merlin D, Bado A (2002) PepT1-mediated fMLP transport induces intestinal inflammation in vivo. Am J Physiol Cell Physiol 283: C1795–1800. 12419711 10.1152/ajpcell.00186.2002

[3] Buyse M, Charrier L, Sitaraman S, Gewirtz A, Merlin D (2003) Interferon-gamma increases hPepT1-mediated uptake of di-tripeptides including the bacterial tripeptide fMLP in polarized intestinal epithelia. Am J Pathol 163: 1969–1977. 14578196 10.1016/s0002-9440(10)63555-9 PMC1892428

[4] Merlin D, Si-Tahar M, Sitaraman SV, Eastburn K, Williams I, et al (2001) Colonic epithelial hPepT1 expression occurs in inflammatory bowel disease: transport of bacterial peptides influences expression of MHC class 1 molecules. Gastroenterology 120: 1666–1679. 11375948 10.1053/gast.2001.24845

[5] Ziegler TR, Fernandez-Estivariz C, Gu LH, Bazargan N, Umeakunne K, et al (2002) Distribution of the H+/peptide transporter PepT1 in human intestine: up-regulated expression in the colonic mucosa of patients with short-bowel syndrome. Am J Clin Nutr 75: 922–930. 11976168 10.1093/ajcn/75.5.922

[6] Wojtal KA, Eloranta JJ, Hruz P, Gutmann H, Drewe J, et al (2009) Changes in mRNA expression levels of solute carrier transporters in inflammatory bowel disease patients. Drug metabolism and disposition: the biological fate of chemicals 37: 1871–1877. 19487253 10.1124/dmd.109.027367

[7] Wang P LY, Wen Y, Yu DY, Ge L, Dong WR, et al (2013) IL-16 Induces Intestinal Inflammation via PepT1 Upregulation in a Pufferfish Model: New Insights into the Molecular Mechanism of Inflammatory Bowel Disease. journal of immunology 1413–1427. 10.4049/jimmunol.1202598 23817423

[8] Zucchelli M, Torkvist L, Bresso F, Halfvarson J, Hellquist A, et al (2009) PepT1 oligopeptide transporter (SLC15A1) gene polymorphism in inflammatory bowel disease. Inflamm Bowel Dis 15: 1562–1569. 19462432 10.1002/ibd.20963

[9] Ayyadurai S, Charania MA, Xiao B, Viennois E, Merlin D (2013) PepT1 expressed in immune cells has an important role in promoting the immune response during experimentally induced colitis. Lab Invest 93: 888–899. 23797361 10.1038/labinvest.2013.77

[10] Dalmasso G, Nguyen HT, Ingersoll SA, Ayyadurai S, Laroui H, et al (2011) The PepT1-NOD2 signaling pathway aggravates induced colitis in mice. Gastroenterology 141: 1334–1345. 21762661 10.1053/j.gastro.2011.06.080 PMC3186842

[11] Yan Y, Kolachala V, Dalmasso G, Nguyen H, Laroui H, et al (2009) Temporal and spatial analysis of clinical and molecular parameters in dextran sodium sulfate induced colitis. PLoS One 4: e6073. 19562033 10.1371/journal.pone.0006073 PMC2698136

[12] Dalmasso G, Charrier-Hisamuddin L, Nguyen HT, Yan Y, Sitaraman S, et al (2008) PepT1-mediated tripeptide KPV uptake reduces intestinal inflammation. Gastroenterology 134: 166–178. 18061177 10.1053/j.gastro.2007.10.026 PMC2431115

[13] Laroui H, Dalmasso G, Nguyen HT, Yan Y, Sitaraman SV, et al (2010) Drug-loaded nanoparticles targeted to the colon with polysaccharide hydrogel reduce colitis in a mouse model. Gastroenterology 138: 843–853 e841–842. 19909746 10.1053/j.gastro.2009.11.003

[14] Laroui H, Ingersoll SA, Liu HC, Baker MT, Ayyadurai S, et al (2012) Dextran sodium sulfate (DSS) induces colitis in mice by forming nano-lipocomplexes with medium-chain-length fatty acids in the colon. PLoS One 7: e32084. 22427817 10.1371/journal.pone.0032084 PMC3302894

[15] Lee RC, Feinbaum RL, Ambros V (1993) The C. elegans heterochronic gene lin-4 encodes small RNAs with antisense complementarity to lin-14. Cell 75: 843–854. 8252621 10.1016/0092-8674(93)90529-y

[16] Wightman B, Ha I, Ruvkun G (1993) Posttranscriptional regulation of the heterochronic gene lin-14 by lin-4 mediates temporal pattern formation in C. elegans. Cell 75: 855–862. 8252622 10.1016/0092-8674(93)90530-4

[17] Wu WK, Law PT, Lee CW, Cho CH, Fan D, et al (2011) MicroRNA in colorectal cancer: from benchtop to bedside. Carcinogenesis 32: 247–253. 21081475 10.1093/carcin/bgq243

[18] Ambros V (2001) microRNAs: tiny regulators with great potential. Cell 107: 823–826. 11779458 10.1016/s0092-8674(01)00616-x

[19] Bartel DP (2004) MicroRNAs: genomics, biogenesis, mechanism, and function. Cell 116: 281–297. 14744438 10.1016/s0092-8674(04)00045-5

[20] Davis BN, Hata A (2009) Regulation of MicroRNA Biogenesis: A miRiad of mechanisms. Cell communication and signaling : CCS 7: 18. 19664273 10.1186/1478-811X-7-18 PMC3224893

[21] Lim LP, Lau NC, Garrett-Engele P, Grimson A, Schelter JM, et al (2005) Microarray analysis shows that some microRNAs downregulate large numbers of target mRNAs. Nature 433: 769–773. 15685193 10.1038/nature03315

[22] Baek D, Villen J, Shin C, Camargo FD, Gygi SP, et al (2008) The impact of microRNAs on protein output. Nature 455: 64–71. 18668037 10.1038/nature07242 PMC2745094

[23] Lewis BP, Shih IH, Jones-Rhoades MW, Bartel DP, Burge CB (2003) Prediction of mammalian microRNA targets. Cell 115: 787–798. 14697198 10.1016/s0092-8674(03)01018-3

[24] Lewis BP, Burge CB, Bartel DP (2005) Conserved seed pairing, often flanked by adenosines, indicates that thousands of human genes are microRNA targets. Cell 120: 15–20. 15652477 10.1016/j.cell.2004.12.035

[25] Taganov KD, Boldin MP, Baltimore D (2007) MicroRNAs and immunity: tiny players in a big field. Immunity 26: 133–137. 17307699 10.1016/j.immuni.2007.02.005

[26] Stefani G, Slack FJ (2008) Small non-coding RNAs in animal development. Nature reviews Molecular cell biology 9: 219–230. 18270516 10.1038/nrm2347

[27] Bueno MJ, Perez de Castro I, Malumbres M (2008) Control of cell proliferation pathways by microRNAs. Cell Cycle 7: 3143–3148. 18843198 10.4161/cc.7.20.6833

[28] Dalmasso G, Nguyen HT, Yan Y, Laroui H, Srinivasan S, et al (2010) MicroRNAs determine human intestinal epithelial cell fate. Differentiation 80: 147–154. 20638171 10.1016/j.diff.2010.06.005 PMC2943016

[29] McKenna LB, Schug J, Vourekas A, McKenna JB, Bramswig NC, et al (2010) MicroRNAs control intestinal epithelial differentiation, architecture, and barrier function. Gastroenterology 139: 1654–1664, 1664 e1651. 20659473 10.1053/j.gastro.2010.07.040 PMC3156097

[30] Dalal SR, Kwon JH (2010) The Role of MicroRNA in Inflammatory Bowel Disease. Gastroenterology & hepatology 6: 714–722. 21437020 PMC3033542

[31] Pekow JR, Kwon JH (2012) MicroRNAs in inflammatory bowel disease. Inflammatory bowel diseases 18: 187–193. 21425211 10.1002/ibd.21691 PMC4169145

[32] Wu F, Zhang S, Dassopoulos T, Harris ML, Bayless TM, et al (2010) Identification of microRNAs associated with ileal and colonic Crohn's disease. Inflammatory bowel diseases 16: 1729–1738. 20848482 10.1002/ibd.21267 PMC2946509

[33] Bartels CL, Tsongalis GJ (2009) MicroRNAs: novel biomarkers for human cancer. Clinical chemistry 55: 623–631. 19246618 10.1373/clinchem.2008.112805

[34] Bartels CL, Tsongalis GJ (2010) [MicroRNAs: novel biomarkers for human cancer]. Annales de biologie clinique 68: 263–272. 20478768 10.1684/abc.2010.0429

[35] Nguyen HT, Dalmasso G, Yan Y, Laroui H, Dahan S, et al (2010) MicroRNA-7 modulates CD98 expression during intestinal epithelial cell differentiation. J Biol Chem 285: 1479–1489. 19892711 10.1074/jbc.M109.057141 PMC2801273

[36] Lagos-Quintana M, Rauhut R, Yalcin A, Meyer J, Lendeckel W, et al (2002) Identification of tissue-specific microRNAs from mouse. Current biology : CB 12: 735–739. 12007417 10.1016/s0960-9822(02)00809-6

[37] Charania MA, Ayyadurai S, Ingersoll SA, Xiao B, Viennois E, et al (2012) Intestinal epithelial CD98 synthesis specifically modulates expression of colonic microRNAs during colitis. Am J Physiol Gastrointest Liver Physiol 302: G1282–1291. 22499850 10.1152/ajpgi.00401.2011 PMC3378169

[38] Viennois E, Chen F, Laroui H, Baker MT, Merlin D (2013) Dextran sodium sulfate inhibits the activities of both polymerase and reverse transcriptase: lithium chloride purification, a rapid and efficient technique to purify RNA. BMC Res Notes 6: 360. 24010775 10.1186/1756-0500-6-360 PMC3847706

[39] Krek A, Grun D, Poy MN, Wolf R, Rosenberg L, et al (2005) Combinatorial microRNA target predictions. Nat Genet 37: 495–500. 15806104 10.1038/ng1536

[40] Griffiths-Jones S, Saini HK, van Dongen S, Enright AJ (2008) miRBase: tools for microRNA genomics. Nucleic Acids Res 36: D154–158. 17991681 10.1093/nar/gkm952 PMC2238936

[41] Grimson A, Farh KK, Johnston WK, Garrett-Engele P, Lim LP, et al (2007) MicroRNA targeting specificity in mammals: determinants beyond seed pairing. Mol Cell 27: 91–105. 17612493 10.1016/j.molcel.2007.06.017 PMC3800283

[42] Bussey KJ, Kane D, Sunshine M, Narasimhan S, Nishizuka S, et al (2003) MatchMiner: a tool for batch navigation among gene and gene product identifiers. Genome biology 4: R27. 12702208 10.1186/gb-2003-4-4-r27 PMC154578

[43] Huang da W, Sherman BT, Lempicki RA (2009) Systematic and integrative analysis of large gene lists using DAVID bioinformatics resources. Nature protocols 4: 44–57. 19131956 10.1038/nprot.2008.211

[44] Kosaka N, Iguchi H, Yoshioka Y, Hagiwara K, Takeshita F, et al (2012) Competitive interactions of cancer cells and normal cells via secretory microRNAs. The Journal of biological chemistry 287: 1397–1405. 22123823 10.1074/jbc.M111.288662 PMC3256909

[45] Kosaka N, Iguchi H, Yoshioka Y, Takeshita F, Matsuki Y, et al (2010) Secretory mechanisms and intercellular transfer of microRNAs in living cells. The Journal of biological chemistry 285: 17442–17452. 20353945 10.1074/jbc.M110.107821 PMC2878508

[46] Wu F, Zikusoka M, Trindade A, Dassopoulos T, Harris ML, et al (2008) MicroRNAs are differentially expressed in ulcerative colitis and alter expression of macrophage inflammatory peptide-2 alpha. Gastroenterology 135: 1624–1635 e1624. 18835392 10.1053/j.gastro.2008.07.068

[47] Wu F, Zhang S, Dassopoulos T, Harris ML, Bayless TM, et al (2010) Identification of microRNAs associated with ileal and colonic Crohn's disease. Inflamm Bowel Dis 16: 1729–1738. 20848482 10.1002/ibd.21267 PMC2946509

[48] Li J, Aderem A (1992) MacMARCKS, a novel member of the MARCKS family of protein kinase C substrates. Cell 70: 791–801. 1516135 10.1016/0092-8674(92)90312-z

[49] Fang K, Bruce M, Pattillo CB, Zhang S, Stone R 2nd, et al (2011) Temporal genomewide expression profiling of DSS colitis reveals novel inflammatory and angiogenesis genes similar to ulcerative colitis. Physiological genomics 43: 43–56. 20923862 10.1152/physiolgenomics.00138.2010 PMC3026350

[50] Saban R, D'Andrea MR, Andrade-Gordon P, Derian CK, Dozmorov I, et al (2007) Regulatory network of inflammation downstream of proteinase-activated receptors. BMC physiology 7: 3. 17397547 10.1186/1472-6793-7-3 PMC1853107

[51] Wang J, Jarrett J, Huang CC, Satcher RL Jr, Levenson AS (2007) Identification of estrogen-responsive genes involved in breast cancer metastases to the bone. Clinical & experimental metastasis 24: 411–422. 17593529 10.1007/s10585-007-9078-6

[52] Valadi H, Ekstrom K, Bossios A, Sjostrand M, Lee JJ, et al (2007) Exosome-mediated transfer of mRNAs and microRNAs is a novel mechanism of genetic exchange between cells. Nat Cell Biol 9: 654–659. 17486113 10.1038/ncb1596

[53] Mittelbrunn M, Gutierrez-Vazquez C, Villarroya-Beltri C, Gonzalez S, Sanchez-Cabo F, et al (2011) Unidirectional transfer of microRNA-loaded exosomes from T cells to antigen-presenting cells. Nat Commun 2: 282. 21505438 10.1038/ncomms1285 PMC3104548

[54] Montecalvo A, Larregina AT, Shufesky WJ, Stolz DB, Sullivan ML, et al (2012) Mechanism of transfer of functional microRNAs between mouse dendritic cells via exosomes. Blood 119: 756–766. 22031862 10.1182/blood-2011-02-338004 PMC3265200

[55] Baj-Krzyworzeka M, Szatanek R, Weglarczyk K, Baran J, Urbanowicz B, et al (2006) Tumour-derived microvesicles carry several surface determinants and mRNA of tumour cells and transfer some of these determinants to monocytes. Cancer Immunol Immunother 55: 808–818. 16283305 10.1007/s00262-005-0075-9 PMC11030663

[56] Ehnfors J, Kost-Alimova M, Persson NL, Bergsmedh A, Castro J, et al (2009) Horizontal transfer of tumor DNA to endothelial cells in vivo. Cell Death Differ 16: 749–757. 19219067 10.1038/cdd.2009.7

[57] Furuta K, Lichtenberger R, Helariutta Y (2012) The role of mobile small RNA species during root growth and development. Current opinion in cell biology 24: 211–216. 22227227 10.1016/j.ceb.2011.12.005

[58] Jin XF, Wu N, Wang L, Li J (2013) Circulating microRNAs: a novel class of potential biomarkers for diagnosing and prognosing central nervous system diseases. Cell Mol Neurobiol 33: 601–613. 23633081 10.1007/s10571-013-9940-9 PMC11497935

[59] Lasser C (2013) Identification and analysis of circulating exosomal microRNA in human body fluids. Methods Mol Biol 1024: 109–128. 23719946 10.1007/978-1-62703-453-1_9

[60] Luo X, Stock C, Burwinkel B, Brenner H (2013) Identification and evaluation of plasma MicroRNAs for early detection of colorectal cancer. PLoS One 8: e62880. 23690963 10.1371/journal.pone.0062880 PMC3653912

[61] Moldovan L, Batte K, Wang Y, Wisler J, Piper M (2013) Analyzing the circulating microRNAs in exosomes/extracellular vesicles from serum or plasma by qRT-PCR. Methods Mol Biol 1024: 129–145. 23719947 10.1007/978-1-62703-453-1_10 PMC3923604

[62] Hannafon BN, Ding WQ (2013) Intercellular Communication by Exosome-Derived microRNAs in Cancer. Int J Mol Sci 14: 14240–14269. 23839094 10.3390/ijms140714240 PMC3742242

[63] Chen X, Liang H, Zhang J, Zen K, Zhang CY (2012) Secreted microRNAs: a new form of intercellular communication. Trends Cell Biol 22: 125–132. 22260888 10.1016/j.tcb.2011.12.001

[64] Muralidharan-Chari V, Clancy JW, Sedgwick A, D'Souza-Schorey C (2010) Microvesicles: mediators of extracellular communication during cancer progression. J Cell Sci 123: 1603–1611. 20445011 10.1242/jcs.064386 PMC2864708

[65] Chatterjee S, Fasler M, Bussing I, Grosshans H (2011) Target-mediated protection of endogenous microRNAs in C. elegans. Dev Cell 20: 388–396. 21397849 10.1016/j.devcel.2011.02.008

[66] Dame MK, Bhagavathula N, Mankey C, DaSilva M, Paruchuri T, et al (2010) Human colon tissue in organ culture: preservation of normal and neoplastic characteristics. In Vitro Cell Dev Biol Anim 46: 114–122. 19915935 10.1007/s11626-009-9247-9 PMC2889208

[67] Nguyen HT, Dalmasso G, Powell KR, Yan Y, Bhatt S, et al (2009) Pathogenic bacteria induce colonic PepT1 expression: an implication in host defense response. Gastroenterology 137: 1435–1447 e1431-1432. 19549526 10.1053/j.gastro.2009.06.043 PMC2757477

[68] Charania MA, Laroui H, Liu H, Viennois E, Ayyadurai S, et al (2013) Intestinal epithelial CD98 directly modulates the innate host response to enteric bacterial pathogens. Infect Immun 81: 923–934. 23297381 10.1128/IAI.01388-12 PMC3584858

