# Supplementary material for: Colonic miRNA Expression/Secretion, Regulated by Intestinal Epithelial PepT1, Plays an Important Role in Cell-to-Cell Communication during Colitis
Source: PLoS One. 2014 Feb 19;9(2):e87614. doi: 10.1371/journal.pone.0087614 (PMC3929505; doi:10.1371/journal.pone.0087614)
Supplement: Table S1 — List of miRNAs differentially expressed in microRNA microarray. The transcripts listed in the table having a statistically significant p-value of p<0.1 and having a signal of >500. (PDF) [file pone.0087614.s001.pdf]

Table S1

No.	Reporter Name	p-value	Villin-hPepT1 H2O Mean	Villin-hPepT1 DSS Mean	FVB/N WT H2O Mean	FVB/N WT DSS Mean
1	mmu-miR-132	1.07E-08	146	1,196	190	575
2	mmu-miR-1937c	2.09E-05	17	385	136	1,810
3	mmu-miR-762	3.73E-05	1,506	10,682	3,312	4,672
4	mmu-miR-200b	2.20E-04	6,182	1,217	6,829	6,328
5	mmu-miR-429	6.66E-04	486	83	710	514
6	mmu-miR-1937b	7.18E-04	2,288	15,875	6,408	18,278
7	mmu-miR-23b	9.14E-04	10,593	3,402	8,243	4,658
8	mmu-miR-1937a	9.83E-04	2,108	17,093	6,412	18,528
9	mmu-miR-199a-3p	1.11E-03	614	267	239	256
10	mmu-miR-23a	1.37E-03	9,029	2,797	6,877	3,608
11	mmu-miR-3077	2.05E-03	270	3,636	1,089	1,293
12	mmu-miR-1934	2.39E-03	145	862	82	91
13	mmu-let-7a	2.64E-03	23,694	15,560	21,543	19,807
14	mmu-miR-2145	2.87E-03	418	2,100	809	737
15	mmu-let-7c	9.39E-03	24,980	16,533	23,931	21,005
