# Supplementary material for: Colonic miRNA Expression/Secretion, Regulated by Intestinal Epithelial PepT1, Plays an Important Role in Cell-to-Cell Communication during Colitis
Source: PLoS One. 2014 Feb 19;9(2):e87614. doi: 10.1371/journal.pone.0087614 (PMC3929505; doi:10.1371/journal.pone.0087614)
Supplement: Table S3 — DAVID functional annotation clustering of villin-hPepT1 and FVB WT at basal level. The common obtained from the matchminer software proteins were clustered into 30 groups. (PDF) [file pone.0087614.s003.pdf]

DAVID Bioinformatics Resources 6.7
National Institute of Allergy and Infectious Diseases (NIAID), NIH

Functional Annotation Clustering

Help and Manual

Current Gene List: List_1
Current Background: Mus musculus
453 DAVID IDs

Options **Classification Stringency** Medium

Rerun using options | Create Sublist

30 Cluster(s)

Download File

	Category	Term			Count	P_Value	Benjamini
**Annotation Cluster 1**		**Enrichment Score: 4.77**	G		Count	P_Value	Benjamini
☐	SP_PIR_KEYWORDS	transcription regulation	RT		72	1.2E-7	1.9E-5
☐	SP_PIR_KEYWORDS	Transcription	RT		72	1.6E-5	1.7E-3
☐	SP_PIR_KEYWORDS	nucleus	RT		128	5.1E-5	4.0E-3
☐	SP_PIR_KEYWORDS	dna-binding	RT		54	9.0E-4	2.6E-2
**Annotation Cluster 2**		**Enrichment Score: 3.31**	G		Count	P_Value	Benjamini
☐	SP_PIR_KEYWORDS	isopeptide bond	RT		18	4.6E-4	2.1E-2
☐	SP_PIR_KEYWORDS	ubl conjugation	RT		27	5.0E-4	2.0E-2
☐	UP_SEQ_FEATURE	cross-link:Glycyl lysine isopeptide (Lys-Gly) (interchain with G-Cter in SUMO)	RT		10	5.1E-4	1.4E-1
**Annotation Cluster 3**		**Enrichment Score: 2.17**	G		Count	P_Value	Benjamini
☐	SP_PIR_KEYWORDS	nucleotide-binding	RT		64	1.5E-4	9.6E-3
☐	SP_PIR_KEYWORDS	atp-binding	RT		51	6.8E-4	2.1E-2
☐	SP_PIR_KEYWORDS	transferase	RT		53	1.1E-3	2.8E-2
☐	SP_PIR_KEYWORDS	kinase	RT		28	1.4E-2	1.9E-1
☐	UP_SEQ_FEATURE	domain:Protein kinase	RT		22	1.5E-2	6.9E-1
☐	UP_SEQ_FEATURE	binding site:ATP	RT		24	1.8E-2	7.2E-1
☐	UP_SEQ_FEATURE	nucleotide phosphate-binding region:ATP	RT		35	2.4E-2	7.5E-1
☐	SP_PIR_KEYWORDS	serine/threonine-protein kinase	RT		17	2.6E-2	2.3E-1
☐	UP_SEQ_FEATURE	active site:Proton acceptor	RT		24	1.1E-1	9.3E-1
**Annotation Cluster 4**		**Enrichment Score: 2.05**	G		Count	P_Value	Benjamini
☐	SP_PIR_KEYWORDS	golgi apparatus	RT		28	5.0E-4	1.8E-2
☐	UP_SEQ_FEATURE	topological domain:Lumenal	RT		20	1.4E-2	7.2E-1
☐	SP_PIR_KEYWORDS	glycosyltransferase	RT		11	2.4E-2	2.3E-1
☐	SP_PIR_KEYWORDS	Signal-anchor	RT		17	3.9E-2	2.9E-1
**Annotation Cluster 5**		**Enrichment Score: 2**	G		Count	P_Value	Benjamini
☐	UP_SEQ_FEATURE	domain:W2	RT		5	1.6E-5	2.3E-2
☐	SP_PIR_KEYWORDS	Initiation factor	RT		4	1.5E-1	6.0E-1
☐	SP_PIR_KEYWORDS	protein biosynthesis	RT		5	4.4E-1	8.7E-1
**Annotation Cluster 6**		**Enrichment Score: 1.83**	G		Count	P_Value	Benjamini
☐	UP_SEQ_FEATURE	repeat:LDL-receptor class B 5	RT		5	2.0E-4	9.3E-2
☐	UP_SEQ_FEATURE	repeat:LDL-receptor class B 1	RT		5	2.8E-4	9.8E-2
☐	UP_SEQ_FEATURE	repeat:LDL-receptor class B 2	RT		5	2.8E-4	9.8E-2
☐	UP_SEQ_FEATURE	repeat:LDL-receptor class B 3	RT		5	2.8E-4	9.8E-2
☐	UP_SEQ_FEATURE	repeat:LDL-receptor class B 4	RT		5	2.8E-4	9.8E-2
☐	UP_SEQ_FEATURE	repeat:LDL-receptor class B 6	RT		4	1.4E-3	2.5E-1
☐	UP_SEQ_FEATURE	repeat:LDL-receptor class B 10	RT		3	9.7E-3	7.0E-1
☐	UP_SEQ_FEATURE	repeat:LDL-receptor class B 14	RT		3	9.7E-3	7.0E-1
☐	UP_SEQ_FEATURE	repeat:LDL-receptor class B 13	RT		3	9.7E-3	7.0E-1

☐	UP_SEQ_FEATURE	repeat:LDL-receptor class B 12	RT			3	9.7E-3	7.0E-1
☐	UP_SEQ_FEATURE	repeat:LDL-receptor class B 11	RT			3	9.7E-3	7.0E-1
☐	UP_SEQ_FEATURE	repeat:LDL-receptor class B 18	RT			3	9.7E-3	7.0E-1
☐	UP_SEQ_FEATURE	repeat:LDL-receptor class B 17	RT			3	9.7E-3	7.0E-1
☐	UP_SEQ_FEATURE	repeat:LDL-receptor class B 16	RT			3	9.7E-3	7.0E-1
☐	UP_SEQ_FEATURE	repeat:LDL-receptor class B 15	RT			3	9.7E-3	7.0E-1
☐	UP_SEQ_FEATURE	repeat:LDL-receptor class B 19	RT			3	9.7E-3	7.0E-1
☐	UP_SEQ_FEATURE	repeat:LDL-receptor class B 20	RT			3	9.7E-3	7.0E-1
☐	UP_SEQ_FEATURE	repeat:LDL-receptor class B 9	RT			3	9.7E-3	7.0E-1
☐	UP_SEQ_FEATURE	repeat:LDL-receptor class B 7	RT			3	1.3E-2	7.3E-1
☐	UP_SEQ_FEATURE	repeat:LDL-receptor class B 8	RT			3	1.3E-2	7.3E-1
☐	UP_SEQ_FEATURE	domain:LDL-receptor class A 3	RT			4	1.5E-2	7.1E-1
☐	UP_SEQ_FEATURE	domain:LDL-receptor class A 8	RT			3	1.7E-2	7.3E-1
☐	UP_SEQ_FEATURE	domain:LDL-receptor class A 1	RT			4	2.2E-2	7.6E-1
☐	UP_SEQ_FEATURE	domain:LDL-receptor class A 2	RT			4	2.4E-2	7.3E-1
☐	UP_SEQ_FEATURE	domain:LDL-receptor class A 7	RT			3	2.7E-2	7.3E-1
☐	UP_SEQ_FEATURE	domain:LDL-receptor class A 6	RT			3	2.7E-2	7.3E-1
☐	UP_SEQ_FEATURE	domain:EGF-like 3	RT			6	3.0E-2	7.5E-1
☐	UP_SEQ_FEATURE	domain:LDL-receptor class A 5	RT			3	3.3E-2	7.7E-1
☐	UP_SEQ_FEATURE	domain:EGF-like 4	RT			5	4.2E-2	8.3E-1
☐	UP_SEQ_FEATURE	domain:LDL-receptor class A 4	RT			3	5.1E-2	8.6E-1
☐	UP_SEQ_FEATURE	short sequence motif:Endocytosis signal	RT			3	5.1E-2	8.6E-1
☐	UP_SEQ_FEATURE	domain:EGF-like 1	RT			7	6.1E-2	8.7E-1
☐	UP_SEQ_FEATURE	domain:EGF-like 6	RT			4	7.3E-2	9.0E-1
☐	SP_PIR_KEYWORDS	Endocytosis	RT			6	7.6E-2	4.2E-1
☐	SP_PIR_KEYWORDS	egf-like domain	RT			10	9.3E-2	4.8E-1
☐	UP_SEQ_FEATURE	domain:EGF-like 7	RT			3	1.4E-1	9.5E-1
☐	UP_SEQ_FEATURE	domain:EGF-like 8	RT			3	1.5E-1	9.6E-1
☐	UP_SEQ_FEATURE	domain:EGF-like 5	RT			3	2.7E-1	9.9E-1
☐	UP_SEQ_FEATURE	domain:EGF-like 2	RT			4	3.5E-1	1.0E0
☐	UP_SEQ_FEATURE	domain:EGF-like 2; calcium-binding	RT			3	4.0E-1	1.0E0
	**Annotation Cluster 7**	**Enrichment Score: 1.56**	G			**Count**	**P_Value**	**Benjamini**
☐	SP_PIR_KEYWORDS	nucleotide binding	RT			6	2.0E-2	2.2E-1
☐	SP_PIR_KEYWORDS	P-loop	RT			6	2.1E-2	2.2E-1
☐	SP_PIR_KEYWORDS	GTP binding	RT			4	5.3E-2	3.5E-1
	**Annotation Cluster 8**	**Enrichment Score: 1.37**	G			**Count**	**P_Value**	**Benjamini**
☐	UP_SEQ_FEATURE	calcium-binding region:2	RT			9	8.6E-3	6.9E-1
☐	UP_SEQ_FEATURE	calcium-binding region:1	RT			9	1.4E-2	7.1E-1
☐	UP_SEQ_FEATURE	domain:EF-hand 2	RT			10	2.6E-2	7.3E-1
☐	UP_SEQ_FEATURE	domain:EF-hand 1	RT			9	6.3E-2	8.8E-1
☐	UP_SEQ_FEATURE	domain:EF-hand 3	RT			5	1.7E-1	9.7E-1
☐	SP_PIR_KEYWORDS	calcium	RT			23	1.8E-1	6.5E-1
	**Annotation Cluster 9**	**Enrichment Score: 1.35**	G			**Count**	**P_Value**	**Benjamini**
☐	SP_PIR_KEYWORDS	cell division	RT			13	2.3E-2	2.3E-1
☐	SP_PIR_KEYWORDS	cell cycle	RT			19	2.6E-2	2.2E-1
☐	SP_PIR_KEYWORDS	mitosis	RT			8	1.5E-1	6.0E-1
	**Annotation Cluster 10**	**Enrichment Score: 1.12**	G			**Count**	**P_Value**	**Benjamini**
☐	UP_SEQ_FEATURE	short sequence motif:DEAD box	RT			4	4.4E-2	8.3E-1
☐	UP_SEQ_FEATURE	short sequence motif:Q motif	RT			4	5.5E-2	8.7E-1
☐	UP_SEQ_FEATURE	domain:Helicase C-terminal	RT			6	7.7E-2	9.1E-1
☐	UP_SEQ_FEATURE	domain:Helicase ATP-binding	RT			6	8.6E-2	9.1E-1

						Count	P_Value	Benjamini
	SP_PIR_KEYWORDS	helicase	RT			6	1.7E-1	6.2E-1
	Annotation Cluster 11	Enrichment Score: 1.1	G			Count	P_Value	Benjamini
	UP_SEQ_FEATURE	domain:WW 3	RT			3	1.7E-2	7.3E-1
	UP_SEQ_FEATURE	domain:WW 2	RT			3	1.7E-1	9.7E-1
	UP_SEQ_FEATURE	domain:WW 1	RT			3	1.7E-1	9.7E-1
	Annotation Cluster 12	Enrichment Score: 0.99	G			Count	P_Value	Benjamini
	SP_PIR_KEYWORDS	lipoprotein	RT			24	1.8E-2	2.2E-1
	SP_PIR_KEYWORDS	palmitate	RT			10	6.4E-2	4.0E-1
	UP_SEQ_FEATURE	lipid moiety-binding region:S-palmitoyl cysteine	RT			9	9.2E-2	9.1E-1
	SP_PIR_KEYWORDS	transducer	RT			9	1.0E0	1.0E0
	Annotation Cluster 13	Enrichment Score: 0.92	G			Count	P_Value	Benjamini
	UP_SEQ_FEATURE	domain:IQ 3	RT			3	5.8E-2	8.8E-1
	UP_SEQ_FEATURE	domain:IQ 2	RT			3	1.7E-1	9.7E-1
	UP_SEQ_FEATURE	domain:IQ 1	RT			3	1.8E-1	9.7E-1
	Annotation Cluster 14	Enrichment Score: 0.89	G			Count	P_Value	Benjamini
	UP_SEQ_FEATURE	metal ion-binding site:Calcium 2; via carbonyl oxygen	RT			4	7.3E-2	9.0E-1
	UP_SEQ_FEATURE	metal ion-binding site:Calcium 3; via carbonyl oxygen	RT			3	1.0E-1	9.3E-1
	UP_SEQ_FEATURE	metal ion-binding site:Calcium 2	RT			4	1.8E-1	9.7E-1
	UP_SEQ_FEATURE	metal ion-binding site:Calcium 3	RT			3	2.0E-1	9.8E-1
	Annotation Cluster 15	Enrichment Score: 0.63	G			Count	P_Value	Benjamini
	UP_SEQ_FEATURE	zinc finger region:C2H2-type 7; atypical	RT			3	2.0E-3	3.1E-1
	UP_SEQ_FEATURE	zinc finger region:C2H2-type 3	RT			11	1.3E-1	9.5E-1
	UP_SEQ_FEATURE	zinc finger region:C2H2-type 1	RT			11	1.3E-1	9.5E-1
	UP_SEQ_FEATURE	zinc finger region:C2H2-type 2	RT			11	1.5E-1	9.6E-1
	UP_SEQ_FEATURE	zinc finger region:C2H2-type 6	RT			6	3.7E-1	1.0E0
	UP_SEQ_FEATURE	zinc finger region:C2H2-type 5	RT			7	3.7E-1	1.0E0
	UP_SEQ_FEATURE	zinc finger region:C2H2-type 4	RT			7	4.6E-1	1.0E0
	UP_SEQ_FEATURE	zinc finger region:C2H2-type 10	RT			3	6.9E-1	1.0E0
	UP_SEQ_FEATURE	zinc finger region:C2H2-type 9	RT			3	7.4E-1	1.0E0
	UP_SEQ_FEATURE	zinc finger region:C2H2-type 8	RT			3	8.2E-1	1.0E0
	UP_SEQ_FEATURE	zinc finger region:C2H2-type 7	RT			3	8.5E-1	1.0E0
	Annotation Cluster 16	Enrichment Score: 0.62	G			Count	P_Value	Benjamini
	UP_SEQ_FEATURE	repeat:ANK 9	RT			4	6.0E-2	8.8E-1
	UP_SEQ_FEATURE	repeat:ANK 8	RT			4	8.3E-2	9.1E-1
	UP_SEQ_FEATURE	repeat:ANK 10	RT			3	1.3E-1	9.5E-1
	UP_SEQ_FEATURE	repeat:ANK 7	RT			4	1.6E-1	9.7E-1
	SP_PIR_KEYWORDS	ank repeat	RT			8	3.0E-1	7.7E-1
	UP_SEQ_FEATURE	repeat:ANK 6	RT			4	3.4E-1	1.0E0
	UP_SEQ_FEATURE	repeat:ANK 1	RT			7	3.9E-1	1.0E0
	UP_SEQ_FEATURE	repeat:ANK 2	RT			7	3.9E-1	1.0E0
	UP_SEQ_FEATURE	repeat:ANK 3	RT			6	4.1E-1	1.0E0
	UP_SEQ_FEATURE	repeat:ANK 4	RT			5	4.3E-1	1.0E0
	UP_SEQ_FEATURE	repeat:ANK 5	RT			4	5.4E-1	1.0E0
	Annotation Cluster 17	Enrichment Score: 0.57	G			Count	P_Value	Benjamini
	UP_SEQ_FEATURE	region of interest:Actin-binding	RT			3	1.3E-1	9.5E-1
	UP_SEQ_FEATURE	domain:Myosin head-like	RT			3	1.8E-1	9.7E-1
	SP_PIR_KEYWORDS	calmodulin-binding	RT			5	2.4E-1	7.2E-1
	SP_PIR_KEYWORDS	myosin	RT			3	3.2E-1	7.9E-1
	SP_PIR_KEYWORDS	motor protein	RT			3	8.0E-1	9.8E-1
	Annotation Cluster 18	Enrichment Score: 0.54	G			Count	P_Value	Benjamini
	SP_PIR_KEYWORDS	tyrosine-protein kinase	RT			6	1.5E-1	6.0E-1

UP_SEQ_FEATURE	domain:Fibronectin type-III 2	RT	5	3.9E-1	1.0E0
UP_SEQ_FEATURE	domain:Fibronectin type-III 1	RT	5	4.0E-1	1.0E0
Annotation Cluster 19	Enrichment Score: 0.51	G	Count	P_Value	Benjamini
UP_SEQ_FEATURE	repeat:WD 13	RT	3	8.8E-2	9.1E-1
UP_SEQ_FEATURE	repeat:WD 12	RT	3	1.0E-1	9.3E-1
UP_SEQ_FEATURE	repeat:WD 8	RT	4	1.3E-1	9.5E-1
UP_SEQ_FEATURE	repeat:WD 11	RT	3	1.6E-1	9.6E-1
UP_SEQ_FEATURE	repeat:WD 10	RT	3	1.6E-1	9.6E-1
UP_SEQ_FEATURE	repeat:WD 9	RT	3	2.3E-1	9.9E-1
UP_SEQ_FEATURE	repeat:WD 7	RT	6	2.3E-1	9.9E-1
UP_SEQ_FEATURE	repeat:WD 6	RT	6	4.2E-1	1.0E0
UP_SEQ_FEATURE	repeat:WD 5	RT	6	6.2E-1	1.0E0
SP_PIR_KEYWORDS	wd repeat	RT	7	6.2E-1	9.4E-1
UP_SEQ_FEATURE	repeat:WD 4	RT	6	6.8E-1	1.0E0
UP_SEQ_FEATURE	repeat:WD 3	RT	6	7.3E-1	1.0E0
UP_SEQ_FEATURE	repeat:WD 1	RT	6	7.5E-1	1.0E0
UP_SEQ_FEATURE	repeat:WD 2	RT	6	7.5E-1	1.0E0
Annotation Cluster 20	Enrichment Score: 0.47	G	Count	P_Value	Benjamini
SP_PIR_KEYWORDS	voltage-gated channel	RT	6	2.3E-1	7.2E-1
SP_PIR_KEYWORDS	potassium channel	RT	4	2.4E-1	7.2E-1
SP_PIR_KEYWORDS	potassium transport	RT	5	2.6E-1	7.5E-1
SP_PIR_KEYWORDS	potassium	RT	5	3.2E-1	7.9E-1
SP_PIR_KEYWORDS	ion transport	RT	14	5.6E-1	9.2E-1
SP_PIR_KEYWORDS	ionic channel	RT	8	6.0E-1	9.3E-1
Annotation Cluster 21	Enrichment Score: 0.43	G	Count	P_Value	Benjamini
SP_PIR_KEYWORDS	zinc-finger	RT	35	2.0E-1	6.8E-1
SP_PIR_KEYWORDS	metal-binding	RT	68	4.2E-1	8.6E-1
SP_PIR_KEYWORDS	zinc	RT	46	5.8E-1	9.3E-1
Annotation Cluster 22	Enrichment Score: 0.42	G	Count	P_Value	Benjamini
UP_SEQ_FEATURE	domain:BACK	RT	3	1.6E-1	9.6E-1
UP_SEQ_FEATURE	domain:BTB	RT	5	3.5E-1	1.0E0
UP_SEQ_FEATURE	repeat:Kelch 5	RT	3	4.0E-1	1.0E0
UP_SEQ_FEATURE	repeat:Kelch 4	RT	3	4.3E-1	1.0E0
UP_SEQ_FEATURE	repeat:Kelch 3	RT	3	4.5E-1	1.0E0
UP_SEQ_FEATURE	repeat:Kelch 2	RT	3	4.5E-1	1.0E0
UP_SEQ_FEATURE	repeat:Kelch 1	RT	3	4.5E-1	1.0E0
SP_PIR_KEYWORDS	kelch repeat	RT	3	4.9E-1	9.0E-1
Annotation Cluster 23	Enrichment Score: 0.38	G	Count	P_Value	Benjamini
UP_SEQ_FEATURE	lipid moiety-binding region:GPI-anchor amidated serine	RT	4	1.8E-1	9.7E-1
SP_PIR_KEYWORDS	gpi-anchor	RT	4	5.8E-1	9.3E-1
UP_SEQ_FEATURE	propeptide:Removed in mature form	RT	6	6.9E-1	1.0E0
Annotation Cluster 24	Enrichment Score: 0.36	G	Count	P_Value	Benjamini
SP_PIR_KEYWORDS	protein phosphatase	RT	5	3.3E-1	8.0E-1
UP_SEQ_FEATURE	domain:Tyrosine-protein phosphatase	RT	3	4.2E-1	1.0E0
UP_SEQ_FEATURE	active site:Phosphocysteine intermediate	RT	3	5.9E-1	1.0E0
Annotation Cluster 25	Enrichment Score: 0.34	G	Count	P_Value	Benjamini
SP_PIR_KEYWORDS	glycoprotein	RT	96	2.0E-1	6.8E-1
SP_PIR_KEYWORDS	disulfide bond	RT	63	4.1E-1	8.5E-1
SP_PIR_KEYWORDS	signal	RT	75	4.3E-1	8.7E-1
SP_PIR_KEYWORDS	Secreted	RT	36	5.1E-1	9.0E-1
UP_SEQ_FEATURE	glycosylation site:N-linked (GlcNAc...)	RT	91	5.5E-1	1.0E0
UP_SEQ_FEATURE	disulfide bond	RT	62	6.1E-1	1.0E0

UP_SEQ_FEATURE	signal peptide	RT	75	7.2E-1	1.0E0
**Annotation Cluster 26**	**Enrichment Score: 0.33**	G	**Count**	**P_Value**	**Benjamini**
UP_SEQ_FEATURE	domain:Ig-like C2-type 1	RT	5	4.5E-1	1.0E0
UP_SEQ_FEATURE	domain:Ig-like C2-type 2	RT	5	4.5E-1	1.0E0
SP_PIR_KEYWORDS	Immunoglobulin domain	RT	12	5.0E-1	9.0E-1
**Annotation Cluster 27**	**Enrichment Score: 0.19**	G	**Count**	**P_Value**	**Benjamini**
SP_PIR_KEYWORDS	glycoprotein	RT	96	2.0E-1	6.8E-1
UP_SEQ_FEATURE	glycosylation site:N-linked (GlcNAc...)	RT	91	5.5E-1	1.0E0
SP_PIR_KEYWORDS	membrane	RT	134	5.5E-1	9.2E-1
UP_SEQ_FEATURE	topological domain:Cytoplasmic	RT	67	8.4E-1	1.0E0
UP_SEQ_FEATURE	topological domain:Extracellular	RT	42	9.9E-1	1.0E0
UP_SEQ_FEATURE	transmembrane region	RT	88	9.9E-1	1.0E0
SP_PIR_KEYWORDS	transmembrane	RT	91	1.0E0	1.0E0
**Annotation Cluster 28**	**Enrichment Score: 0.18**	G	**Count**	**P_Value**	**Benjamini**
UP_SEQ_FEATURE	repeat:LRR 11	RT	4	4.1E-1	1.0E0
UP_SEQ_FEATURE	repeat:LRR 10	RT	4	5.1E-1	1.0E0
UP_SEQ_FEATURE	repeat:LRR 13	RT	3	5.1E-1	1.0E0
UP_SEQ_FEATURE	repeat:LRR 12	RT	3	6.2E-1	1.0E0
UP_SEQ_FEATURE	repeat:LRR 5	RT	6	6.2E-1	1.0E0
UP_SEQ_FEATURE	repeat:LRR 9	RT	4	6.3E-1	1.0E0
UP_SEQ_FEATURE	repeat:LRR 8	RT	4	6.8E-1	1.0E0
UP_SEQ_FEATURE	repeat:LRR 4	RT	6	7.0E-1	1.0E0
UP_SEQ_FEATURE	repeat:LRR 6	RT	5	7.2E-1	1.0E0
UP_SEQ_FEATURE	repeat:LRR 3	RT	6	7.8E-1	1.0E0
UP_SEQ_FEATURE	repeat:LRR 7	RT	4	7.9E-1	1.0E0
SP_PIR_KEYWORDS	leucine-rich repeat	RT	6	8.0E-1	9.8E-1
UP_SEQ_FEATURE	repeat:LRR 1	RT	6	8.3E-1	1.0E0
UP_SEQ_FEATURE	repeat:LRR 2	RT	6	8.3E-1	1.0E0
**Annotation Cluster 29**	**Enrichment Score: 0.07**	G	**Count**	**P_Value**	**Benjamini**
UP_SEQ_FEATURE	repeat:TPR 1	RT	3	8.6E-1	1.0E0
UP_SEQ_FEATURE	repeat:TPR 2	RT	3	8.6E-1	1.0E0
SP_PIR_KEYWORDS	tpr repeat	RT	3	8.6E-1	9.9E-1
**Annotation Cluster 30**	**Enrichment Score: 0.01**	G	**Count**	**P_Value**	**Benjamini**
SP_PIR_KEYWORDS	transit peptide	RT	7	9.7E-1	1.0E0
UP_SEQ_FEATURE	transit peptide:Mitochondrion	RT	7	9.8E-1	1.0E0
SP_PIR_KEYWORDS	mitochondrion	RT	11	9.9E-1	1.0E0

161 terms were not clustered.

Please cite *Nature Protocols 2009; 4(1):44 & Genome Biology 2003; 4(5):P3* within any publication that makes use of any methods inspired by **DAVID**.

Term of Service | Contact Us | Site Map
