# Supplementary material for: Colonic miRNA Expression/Secretion, Regulated by Intestinal Epithelial PepT1, Plays an Important Role in Cell-to-Cell Communication during Colitis
Source: PLoS One. 2014 Feb 19;9(2):e87614. doi: 10.1371/journal.pone.0087614 (PMC3929505; doi:10.1371/journal.pone.0087614)
Supplement: Table S4 — DAVID functional annotation clustering of villin-hPepT1 and FVB WT mice treated with DSS. The common proteins obtained from the matchminer software were clustered into 15 groups. (PDF) [file pone.0087614.s004.pdf]

DAVID Bioinformatics Resources 6.7
National Institute of Allergy and Infectious Diseases (NIAID), NIH

Functional Annotation Clustering

Help and Manual

Current Gene List: List_1
Current Background: Mus musculus
196 DAVID IDs

Options   Classification Stringency Medium

Rerun using options   Create Sublist

15 Cluster(s)   Download File

	Annotation Cluster 1	Enrichment Score: 2.96	G		Count	P_Value	Benjamini
☐	SP_PIR_KEYWORDS	transcription regulation	RT		34	7.8E-5	9.1E-3
☐	SP_PIR_KEYWORDS	Transcription	RT		34	9.1E-4	6.8E-2
☐	SP_PIR_KEYWORDS	nucleus	RT		59	1.6E-3	8.8E-2
☐	SP_PIR_KEYWORDS	activator	RT		14	2.0E-3	8.9E-2
☐	SP_PIR_KEYWORDS	dna-binding	RT		26	7.2E-3	2.1E-1
	**Annotation Cluster 2**	**Enrichment Score: 1.92**	**G**		**Count**	**P_Value**	**Benjamini**
☐	SP_PIR_KEYWORDS	isopeptide bond	RT		9	9.5E-3	2.4E-1
☐	SP_PIR_KEYWORDS	ubl conjugation	RT		13	1.0E-2	2.3E-1
☐	UP_SEQ_FEATURE	cross-link:Glycyl lysine isopeptide (Lys-Gly) (interchain with G-Cter in SUMO)	RT		5	1.9E-2	9.6E-1
	**Annotation Cluster 3**	**Enrichment Score: 1.79**	**G**		**Count**	**P_Value**	**Benjamini**
☐	UP_SEQ_FEATURE	calcium-binding region:2	RT		7	1.7E-3	7.6E-1
☐	UP_SEQ_FEATURE	calcium-binding region:1	RT		7	2.6E-3	6.7E-1
☐	UP_SEQ_FEATURE	domain:EF-hand 2	RT		7	1.0E-2	9.5E-1
☐	UP_SEQ_FEATURE	domain:EF-hand 1	RT		6	3.8E-2	9.9E-1
☐	UP_SEQ_FEATURE	domain:EF-hand 3	RT		4	6.8E-2	1.0E0
☐	SP_PIR_KEYWORDS	calcium	RT		12	1.5E-1	6.4E-1
	**Annotation Cluster 4**	**Enrichment Score: 1.12**	**G**		**Count**	**P_Value**	**Benjamini**
☐	SP_PIR_KEYWORDS	potassium transport	RT		5	2.7E-2	3.9E-1
☐	SP_PIR_KEYWORDS	potassium	RT		5	3.7E-2	4.7E-1
☐	SP_PIR_KEYWORDS	potassium channel	RT		4	3.8E-2	4.5E-1
☐	SP_PIR_KEYWORDS	voltage-gated channel	RT		5	5.5E-2	5.0E-1
☐	SP_PIR_KEYWORDS	ion transport	RT		9	2.2E-1	7.5E-1
☐	SP_PIR_KEYWORDS	ionic channel	RT		5	3.9E-1	8.8E-1
	**Annotation Cluster 5**	**Enrichment Score: 0.99**	**G**		**Count**	**P_Value**	**Benjamini**
☐	SP_PIR_KEYWORDS	atp-binding	RT		23	1.8E-2	3.2E-1
☐	SP_PIR_KEYWORDS	nucleotide-binding	RT		26	3.8E-2	4.4E-1
☐	SP_PIR_KEYWORDS	kinase	RT		13	7.3E-2	5.5E-1
☐	SP_PIR_KEYWORDS	transferase	RT		21	9.7E-2	5.9E-1
☐	UP_SEQ_FEATURE	binding site:ATP	RT		11	9.8E-2	1.0E0
☐	UP_SEQ_FEATURE	nucleotide phosphate-binding region:ATP	RT		16	9.9E-2	1.0E0
☐	SP_PIR_KEYWORDS	tyrosine-protein kinase	RT		4	1.3E-1	6.2E-1
☐	UP_SEQ_FEATURE	domain:Protein kinase	RT		9	1.8E-1	1.0E0
☐	SP_PIR_KEYWORDS	serine/threonine-protein kinase	RT		7	2.2E-1	7.5E-1
☐	UP_SEQ_FEATURE	active site:Proton acceptor	RT		9	4.9E-1	1.0E0
	**Annotation Cluster 6**	**Enrichment Score: 0.76**	**G**		**Count**	**P_Value**	**Benjamini**
☐	SP_PIR_KEYWORDS	egf-like domain	RT		6	8.8E-2	5.8E-1
☐	UP_SEQ_FEATURE	domain:EGF-like 3	RT		3	1.8E-1	1.0E0

	UP_SEQ_FEATURE	domain:EGF-like 1	RT		3	3.4E-1	1.0E0
	Annotation Cluster 7	Enrichment Score: 0.68	G		Count	P_Value	Benjamini
	UP_SEQ_FEATURE	repeat:ANK 9	RT		3	5.8E-2	1.0E0
	UP_SEQ_FEATURE	repeat:ANK 8	RT		3	7.4E-2	1.0E0
	UP_SEQ_FEATURE	repeat:ANK 7	RT		3	1.2E-1	1.0E0
	SP_PIR_KEYWORDS	ank repeat	RT		5	2.1E-1	7.4E-1
	UP_SEQ_FEATURE	repeat:ANK 6	RT		3	2.3E-1	1.0E0
	UP_SEQ_FEATURE	repeat:ANK 3	RT		4	2.8E-1	1.0E0
	UP_SEQ_FEATURE	repeat:ANK 5	RT		3	3.5E-1	1.0E0
	UP_SEQ_FEATURE	repeat:ANK 1	RT		4	3.8E-1	1.0E0
	UP_SEQ_FEATURE	repeat:ANK 2	RT		4	3.8E-1	1.0E0
	UP_SEQ_FEATURE	repeat:ANK 4	RT		3	4.3E-1	1.0E0
	Annotation Cluster 8	Enrichment Score: 0.64	G		Count	P_Value	Benjamini
	SP_PIR_KEYWORDS	golgi apparatus	RT		10	1.3E-1	6.3E-1
	SP_PIR_KEYWORDS	Signal-anchor	RT		7	2.6E-1	7.8E-1
	UP_SEQ_FEATURE	topological domain:Lumenal	RT		7	3.4E-1	1.0E0
	Annotation Cluster 9	Enrichment Score: 0.44	G		Count	P_Value	Benjamini
	SP_PIR_KEYWORDS	zinc finger	RT		3	1.5E-1	6.3E-1
	UP_SEQ_FEATURE	zinc finger region:C2H2-type 3	RT		5	3.3E-1	1.0E0
	UP_SEQ_FEATURE	zinc finger region:C2H2-type 1	RT		5	3.3E-1	1.0E0
	UP_SEQ_FEATURE	zinc finger region:C2H2-type 2	RT		5	3.5E-1	1.0E0
	UP_SEQ_FEATURE	zinc finger region:C2H2-type 4	RT		4	4.2E-1	1.0E0
	SP_PIR_KEYWORDS	zinc-finger	RT		14	5.1E-1	9.2E-1
	UP_SEQ_FEATURE	zinc finger region:C2H2-type 5	RT		3	6.4E-1	1.0E0
	Annotation Cluster 10	Enrichment Score: 0.34	G		Count	P_Value	Benjamini
	SP_PIR_KEYWORDS	palmitate	RT		4	3.7E-1	8.8E-1
	SP_PIR_KEYWORDS	lipoprotein	RT		8	4.3E-1	8.9E-1
	UP_SEQ_FEATURE	lipid moiety-binding region:S-palmitoyl cysteine	RT		3	6.0E-1	1.0E0
	Annotation Cluster 11	Enrichment Score: 0.29	G		Count	P_Value	Benjamini
	UP_SEQ_FEATURE	domain:Ig-like C2-type 1	RT		3	4.4E-1	1.0E0
	UP_SEQ_FEATURE	domain:Ig-like C2-type 2	RT		3	4.5E-1	1.0E0
	SP_PIR_KEYWORDS	Immunoglobulin domain	RT		5	6.9E-1	9.7E-1
	Annotation Cluster 12	Enrichment Score: 0.17	G		Count	P_Value	Benjamini
	SP_PIR_KEYWORDS	signal	RT		33	4.9E-1	9.1E-1
	SP_PIR_KEYWORDS	glycoprotein	RT		39	5.4E-1	9.3E-1
	SP_PIR_KEYWORDS	Secreted	RT		15	6.5E-1	9.6E-1
	UP_SEQ_FEATURE	signal peptide	RT		33	6.8E-1	1.0E0
	SP_PIR_KEYWORDS	disulfide bond	RT		24	7.8E-1	9.9E-1
	UP_SEQ_FEATURE	glycosylation site:N-linked (GlcNAc...)	RT		36	8.2E-1	1.0E0
	UP_SEQ_FEATURE	disulfide bond	RT		24	8.4E-1	1.0E0
	Annotation Cluster 13	Enrichment Score: 0.15	G		Count	P_Value	Benjamini
	SP_PIR_KEYWORDS	zinc-finger	RT		14	5.1E-1	9.2E-1
	SP_PIR_KEYWORDS	zinc	RT		18	7.9E-1	9.9E-1
	SP_PIR_KEYWORDS	metal-binding	RT		24	8.9E-1	1.0E0
	Annotation Cluster 14	Enrichment Score: 0.13	G		Count	P_Value	Benjamini
	SP_PIR_KEYWORDS	glycoprotein	RT		39	5.4E-1	9.3E-1
	UP_SEQ_FEATURE	topological domain:Cytoplasmic	RT		32	6.0E-1	1.0E0
	SP_PIR_KEYWORDS	membrane	RT		58	6.1E-1	9.5E-1
	UP_SEQ_FEATURE	glycosylation site:N-linked (GlcNAc...)	RT		36	8.2E-1	1.0E0
	UP_SEQ_FEATURE	topological domain:Extracellular	RT		22	8.3E-1	1.0E0
	UP_SEQ_FEATURE	transmembrane region	RT		41	9.1E-1	1.0E0
	SP_PIR_KEYWORDS	transmembrane	RT		41	1.0E0	1.0E0

	Annotation Cluster 15	Enrichment Score: 0.04	G			Count	P_Value	Benjamini
☐	SP_PIR_KEYWORDS	transit peptide	RT			4	8.7E-1	1.0E0
☐	UP_SEQ_FEATURE	transit peptide:Mitochondrion	RT			4	8.9E-1	1.0E0
☐	SP_PIR_KEYWORDS	mitochondrion	RT			5	9.7E-1	1.0E0

83 terms were not clustered.

Please cite *Nature Protocols 2009; 4(1):44 & Genome Biology 2003; 4(5):P3* within any publication that makes use of any methods inspired by **DAVID.**

Term of Service | Contact Us | Site Map
