# Supplementary material for: Colonic miRNA Expression/Secretion, Regulated by Intestinal Epithelial PepT1, Plays an Important Role in Cell-to-Cell Communication during Colitis
Source: PLoS One. 2014 Feb 19;9(2):e87614. doi: 10.1371/journal.pone.0087614 (PMC3929505; doi:10.1371/journal.pone.0087614)
Supplement: Table S5 — List of primers used in this study. (PDF) [file pone.0087614.s005.pdf]

Table S5

Primer	Sequence	Description
snoRNA234	GGAACTGAATCTAAGTGATTTAACAA	Small RNA 234 forward primer
mmT-miR-132F	TAACAGTCTACAGCCATGGTCG	MiRNA 132 forward primer
mmT-miR-1937cF	ATCCCGGAAGAGCCCCCA	MiRNA 1937c forward primer
mmT-miR-762F	GGGGCTGGGGCCGGGACAGAGC	MiRNA 762 forward primer
mmT-miR-1937aF	AATCCCGGACGAGCCCCCA	MiRNA 1937a forward primer
mmT-miR-1937bF	ATCCCGGACGAGCCCCCA	MiRNA 1937b forward primer
mmT-miR-2145 F	AGCAGGGTCGGGCCTGGTT	MiRNA 2145 forward primer
mmT-miR-200bF	TAATACTGCCTGGTAATGATGA	MiRNA 200bforward primer
mmT-miR-3077F	CTGACTCCCTGCTTCTCCGCAG	MiRNA 3077 forward primer
mmT-miR-429F	TAATACTGTCTGGTAATGCCGT	MiRNA 429 forward primer
mmT-miR-23aF	ATCACATTGCCAGGGATTTCC	MiRNA 23a forward primer
mmT-miR-23bF	ATCACATTGCCAGGGATTACC	MiRNA 23b forward primer
mmT-miR-199a-3p F	ACAGTAGTCTGCACATTGGTTA	MiRNA 199a-3p forward primer
mmT-let-7c F	TGAGGTAGTAGGTTGTATGGTT	MiRNA 7c forward primer
mmT-let-7b F	TGAGGTAGTAGGTTGTGTGGTT	MiRNA 7b forward primer
mmT-miR-1934F	TCTGGTCCCCTGCTTCGTCCTCT	MiRNA 1934 forward primer
36B4 F	TCCAGGCTTTGGGCATCA	36B4 forward primer
36B4 R	CTTTATCAGCTGCACATCACTCAGA	36B4 reverse primer
Marcksl-1 F	AGGAGGGAGGGAGGTCTGTA	Marcksl-1 forward Primer
Marcksl-1 R	GGGGTTTGGCCATTAAAAGT	Marcksl-1 reverse Primer
